# Targeting the NAT10-HDAC4 positive feedback loop counteracts immunosuppression in breast cancer

**DOI:** 10.1186/s13046-025-03638-7

**Published:** 2026-01-07

**Authors:** Xin Ma, Shengye Jin, Xingda Zhang, Liuying Zhao, Haoran Wang, Siyu Liu, Hui Li, Qin Wang, Song Gao, Jianyu Wang, Yajie Gong, Yijun Chu, Crystal Song Zhang, Xi Chen, Da Pang, Cheng Qian, Hao Wu

**Affiliations:** 1 Department of Breast Surgery, Harbin Medical University Cancer Hospital, 150 Haping Road, Harbin, 150081 China; 2 Heilongjiang Clinical Research Center for Breast Cancer, Harbin Medical University Cancer Hospital, Harbin, 150081 China; 3 Translational Medicine Research and Cooperation Center of Northern China, Heilongjiang Academy of Medical Sciences, Harbin, 150081 China; 4 Emory University, College of Arts and Science, Atlanta, GA 30322 US; 5 Department of Breast Surgery, The Second Affiliated Hospital of Harbin Medical University, Harbin, 150086 China

**Keywords:** NAT10, ac4C modification, HDAC4, PD-L1, Immunosuppression

## Abstract

**Background:**

N-acetyltransferase 10 (NAT10) mediated N4-acetylcytidine (ac4C) modification has been implicated in tumor progression; however, the precise role and underlying mechanism of NAT10 in breast cancer progression remain largely undefined.

**Methods:**

The expression and prognostic significance of NAT10 in breast cancer were evaluated using clinical tissue samples and public databases. Functional assays were performed in vitro and in vivo to assess the effects of NAT10 on tumor growth and immune evasion. Mechanistic studies, including RNA immunoprecipitation (RIP), ac4C RNA immunoprecipitation (acRIP), and co-immunoprecipitation (Co-IP), were conducted to elucidate the interaction between NAT10 and histone deacetylase 4 (HDAC4) and their roles in regulating NF-κB signaling and programmed death-ligand 1 (PD-L1) expression.

**Results:**

NAT10 expression was significantly upregulated in breast cancer and correlated with poor patient prognosis. NAT10 mediated ac4C modification enhanced the stability of HDAC4 mRNA, thereby promoting HDAC4 expression. Conversely, HDAC4 stabilized NAT10 protein through post-transcriptional deacetylation, forming a self-reinforcing regulatory loop. Elevated HDAC4 activated the NF-κB signaling pathway, resulting in increased PD-L1 transcription and enhanced immune evasion of breast cancer cells. Inhibition of the NAT10/HDAC4/NF-κB axis markedly reduced PD-L1 expression and restored antitumor immune responses.

**Conclusion:**

Our findings identify a self-reinforcing NAT10/HDAC4 signaling circuit that drives breast cancer progression and immune evasion. Targeting NAT10 represents a promising therapeutic strategy to overcome immunosuppression and improve patient outcomes in breast cancer.

**Supplementary Information:**

The online version contains supplementary material available at 10.1186/s13046-025-03638-7.

## Introduction

Breast cancer is the most prevalent malignancy among women worldwide and is characterized by high incidence and mortality rates [1]. Despite considerable progress in surgery, chemotherapy, radiotherapy, hormone therapy, targeted therapy, and immunotherapy, challenges such as drug resistance, tumor recurrence, and immune evasion remain prominent in breast cancer management [2–5]. Therefore, novel molecular markers and therapeutic targets are urgently needed to improve patient outcomes.

RNA modification plays a critical role in regulating the malignant biological characteristics of tumors and represents a potential target for cancer therapy [6–8]. The recently identified N4-acetylcytidine (ac4C) modification, which occurs in tRNA, rRNA, and mRNA, is highly conserved across species and regulates both mRNA stability and translation efficiency [9–11]. N-acetyltransferase 10 (NAT10), the sole known enzyme catalyzing ac4C modification, belongs to the GCN5-related N-acetyltransferase (GNAT) family and contains both acetyltransferase and RNA binding domains [9, 12]. NAT10 is highly expressed in a variety of cancers, and its expression frequently correlates with poor prognosis [13, 14]. Previous studies have shown that NAT10 mediated ac4C modification enhances the stability and translation efficiency of specific mRNAs, thereby enabling cancer cells to adapt to the dynamic tumor microenvironment and supporting their proliferation, invasion, migration, metastasis, and therapeutic resistance. For example, NAT10 enhances the stability of kinesin family member 23 (KIF23) mRNA via ac4C modification, establishing a NAT10-KIF23-GSK-3β-β-catenin axis that accelerates colorectal cancer progression [15]. In gastric cancer, hypoxia inducible factor 1 (HIF-1) regulates NAT10 transcription, promoting ac4C modification of Septin 9 (SEPT9) mRNA, which activates the HIF-1 pathway and establishes a NAT10/SEPT9/HIF-1 feedback loop that enhances glycolytic dependency and tumor progression [16]. Zhou et al. demonstrated that NAT10 facilitates ac4C modification of integrin beta 5 (ITGB5) mRNA, contributing to tumor invasion and metastasis [17]. In cervical cancer, the NAT10/ac4C/FOXP1 axis promotes glycolysis and sustained lactate secretion, thereby facilitating tumor progression [18]. In bladder cancer, NAT10 mediated ac4C modification markedly increases the stability of DNA damage repair genes, contributing to cisplatin resistance [19].

Given the central role of NAT10 in tumor development, targeting NAT10 or its downstream pathways is considered a promising therapeutic strategy. Inhibiting NAT10 or disrupting its interactions with oncogenes may slow tumor progression and improve the efficacy of existing therapies. Therefore, elucidating the mechanisms by which NAT10 mediated ac4C modification contributes to breast cancer progression may provide novel therapeutic strategies and targets.

In this study, we demonstrated that NAT10 mediated ac4C modification promoted breast cancer progression and correlated with poor prognosis. Mechanistically, NAT10 stabilized the mRNA of HDAC4, a member of the class II histone deacetylase family, through ac4C modification. In turn, HDAC4 maintained NAT10 protein stability through post-transcriptional deacetylation. Furthermore, HDAC4 promoted PD-L1 expression by activating the NF-κB signaling pathway, thereby facilitating immune evasion by breast cancer cells. Inhibition of the NAT10/HDAC4/NF-κB pathway suppressed PD-L1 expression, thereby enhancing antitumor immune responses and reducing immunosuppression in breast cancer. These findings suggest that NAT10 represents a promising therapeutic target to enhance antitumor immunity. Our study provides new insights into the molecular mechanisms of breast cancer progression and identifies potential therapeutic strategies to improve patient outcomes and survival.

## Materials and methods

### Breast tissue specimens

A total of 220 breast cancer tissues and 24 normal breast tissues were collected from patients who underwent surgical resection at Harbin Medical University Cancer Hospital. All samples were fixed in 4% formalin and embedded in paraffin for immunohistochemistry (IHC). The clinicopathological characteristics of these patients are summarized in Table S1. The survival analysis was conducted using follow-up data from breast cancer patients. Overall survival (OS) was defined as the interval between the date of surgery and either death from any cause or the last recorded follow-up. Only patients with complete clinicopathological information and available overall survival data were included in the analysis. In addition, eight paired breast cancer tissues and adjacent normal tissues were snap frozen in liquid nitrogen immediately after resection and stored at − 80 °C for subsequent RNA and protein extraction. Eligible patients were those with a histopathological diagnosis of breast cancer who had not received chemotherapy or radiotherapy prior to surgery. Written informed consent was obtained from each participant before sample collection. The use of human tissue specimens was approved by the Research Ethics Committee of Harbin Medical University Cancer Hospital (Approval No. KY2022-56) and conducted in accordance with institutional clinical research guidelines.

### Cell culture and transfection

Breast cancer cell lines (4T1, BT549, and MDA-MB-231) and 293T cells were obtained from the National Collection of Authenticated Cell Cultures of Chinese Academy of Sciences (Shanghai, China). All cell lines were cultured under standard conditions recommended by the supplier in medium supplemented with 10% fetal bovine serum (FBS, Gibco), 100 U/mL penicillin, and 100 µg/mL streptomycin. 4T1 and 293T cells were cultured in Dulbecco’s Modified Eagle Medium (DMEM; Gibco) at 37 °C with 5% CO₂, whereas BT549 cells were cultured in Roswell Park Memorial Institute 1640 medium (RPMI 1640; Gibco) under the same conditions. MDA-MB-231 cells were cultured in Leibovitz’s L-15 medium (L-15; Gibco) at 37 °C in a no CO₂ incubator.

Cells were seeded in six well plates and transfected with small interfering RNA (siRNA) or plasmids using JetPrime reagent (Polyplus, #101000046, Germany) at 50%-70% confluence according to the manufacturer’s instructions. For lentiviral transduction, polybrene (MilliporeSigma, #TR-1003, USA) was added at 4–6 µg/mL to enhance infection efficiency, and transduced cells were selected with 1 µg/mL puromycin (MilliporeSigma, #540411, USA). The sequences of siRNA, plasmids, and lentivirus based short hairpin RNA (shRNA) are provided in Table S2. The efficiency of knockdown or overexpression was validated by quantitative reverse transcription PCR (qRT-PCR) and Western blot analysis.

### Cell proliferation assay (CCK-8 assay)

Cell proliferation was assessed using the Cell Counting Kit-8 (CCK-8; Meilunbio, #MA0218, China). Briefly, 3000–5000 cells per well were seeded into 96 well plates (200 µL medium per well) and cultured under the indicated conditions. At designated time points (0, 24, 48, 72, and 96 h), 10 µL of CCK-8 reagent was added to each well and incubated at 37 °C for 1–2 h in the dark. Absorbance at 450 nm was measured using a microplate reader. Each condition was tested in six replicate wells, and cell growth curves were generated from the absorbance values.

### Flow cytometry evaluating apoptosis and cell cycle

Flow cytometry was performed to evaluate apoptosis and cell cycle distribution. For apoptosis analysis, cells were harvested, washed twice with cold PBS, and stained with Annexin V-AbFluor™ 488 and propidium iodide (PI) using the Annexin V-AbFluor™ 488/PI Apoptosis Detection Kit (Abbkine, #KTA0002, China), according to the manufacturer’s instructions. After 15 min of incubation at room temperature in the dark, samples were analyzed using a BD Canto II flow cytometer (Thermo Fisher Scientific, USA). Data were processed with FlowJo software to quantify apoptotic cell proportions.

For cell cycle analysis, cells were collected, washed with PBS, and fixed overnight in 70% ethanol at 4 °C. Fixed cells were then treated with 100 µg/mL RNase A for 30 min at 37 °C and stained with PI using the Cell Cycle Detection Kit (Abbkine, #KTA2020, China) according to the manufacturer’s instructions. Samples were analyzed with a BD Canto II flow cytometer, and cell-cycle distribution was quantified using ModFit software.

For cell surface staining of single cell suspensions, cells were first stained with a viability dye. Subsequently, they were incubated with fluorochrome conjugated anti-CD45, anti-CD3, and anti-CD8 antibodies at 4 °C for 30 min. Finally, samples were analyzed using a BD Canto II flow cytometer (Thermo Fisher Scientific, USA). Data analysis was performed with FlowJo software. Detailed information on the antibodies used is provided in Table S3.

### qRT-PCR and RNA decay assay

Total RNA was extracted from breast cancer tissues and cells using TRIzol reagent (Invitrogen, #15596-026, USA) in accordance with the manufacturer’s instructions. RNA was reverse transcribed into complementary DNA (cDNA) using a PrimeScript RT Reagent Kit (TaKaRa, #RR047Q, Japan). Quantitative PCR was performed with SYBR Green Master Mix (Roche, #04913914001, Switzerland) on an ABI 7500 FAST Real-Time PCR System (Applied Biosystems, CA, USA). β-actin served as the internal control. Relative gene expression was calculated using the 2^−ΔΔCt^method. Primer sequences used in this study are listed in Table S2.

For RNA decay analysis, cells were treated with 5 µg/mL actinomycin D (MedChemExpress, #HY-17559, China) for different time periods (0, 0.5, 1, and 1.5 h). Total RNA was extracted at each time point and analyzed by qRT-PCR to determine relative gene expression.

### Western blot and Co-Immunoprecipitation (Co-IP) assays

Cells and tissues were lysed with RIPA lysis buffer (Solarbio, #R0010, China) supplemented with protease and phosphatase inhibitors at 4 °C for 30 min, followed by ultrasonic disruption on ice. Lysates were centrifuged at 12,000 g for 10 min at 4 °C, and the supernatant was collected. Protein concentration was determined using a bicinchoninic acid (BCA) assay kit (Beyotime, #P0010, China). Equal amounts of protein were separated on 8–10% SDS-PAGE gels, depending on molecular weight, and transferred to polyvinylidene fluoride (PVDF) membranes (MilliporeSigma, #03010040001, USA). Membranes were blocked with 5% non-fat milk in TBST (25 mM Tris, 150 mM NaCl, 0.1% Tween-20, pH 7.4) for 1 h at room temperature, then incubated overnight at 4 °C with primary antibodies diluted in TBST. After three washes, membranes were incubated with HRP conjugated secondary antibodies for 1 h at room temperature. Protein bands were visualized using an enhanced chemiluminescence detection system (Tanon 4600, China). The antibodies used in this study are listed in Table S3.

For Co-IP, cell lysates were prepared using IP lysis buffer (Thermo Fisher Scientific, #87787, USA) supplemented with protease and phosphatase inhibitors. Equal amounts of protein were incubated with specific antibodies or control IgG overnight at 4 °C with gentle rotation, followed by incubation with Protein A/G magnetic beads (MedChemExpress, #HY-K0202, China) for 2 h. Beads were washed three times with lysis buffer, and bound proteins were eluted by boiling in SDS loading buffer, followed by Western blot analysis as described above.

For protein half-life assays, cells were treated with 100 µg/mL cycloheximide (MedChemExpress, #HY-12320, China) for the indicated time periods (0, 2, 4, and 6 h). Total protein was extracted and subjected to Western blot analysis.

### N4-acetylcytidine (ac4C) Dot blot

Total RNA was denatured at 95 °C for 3 min, immediately placed on ice, and spotted onto Hybond-N + membranes (Beyotime, #FFN10, China). Membranes were UV-crosslinked at 150 mJ/cm² (254 nm), then blocked with 5% non-fat milk in TBST for 1 h at room temperature. After blocking, membranes were incubated overnight at 4 °C with anti-ac4C antibody (1:250 dilution) diluted in TBST. Following three washes with TBST, membranes were incubated with HRP-conjugated goat anti-rabbit IgG (H + L) for 1 h at room temperature. Signals were detected using an enhanced chemiluminescence detection system (Tanon 4600, China). Methylene blue staining (0.02% in 0.3 M sodium acetate, pH 5.2) was performed as a loading control to visualize total RNA.

### Hematoxylin & Eosin (HE) and immunohistochemistry (IHC) staining

Paraffin-embedded human and mouse tumor tissues were sectioned at 3 μm thickness. Sections were heated at 65 °C for 3 h, deparaffinized in xylene, and rehydrated through graded ethanol. For H&E staining, slides were stained with hematoxylin for 5 min, rinsed in running tap water, and counterstained with eosin for 2 min. Sections were then dehydrated through graded ethanol, cleared in xylene, and mounted with neutral mounting medium. For IHC, antigen retrieval was performed in EDTA buffer (pH 9.0) using a pressure cooker. Endogenous peroxidase activity was blocked with 3% H₂O₂ for 15 min, and nonspecific binding was blocked with 5% bovine serum albumin (BSA) for 30 min at room temperature. Sections were incubated overnight at 4 °C with the indicated primary antibodies. After washing, sections were incubated with goat anti-rabbit IgG secondary antibody for 1 h at room temperature. Visualization was achieved using DAB solution (ZSGB-BIO, #ZLI-9018, China), and nuclei were counterstained with hematoxylin. Stained slides were independently evaluated by two pathologists. Final IHC scores were calculated as the product of staining intensity and extent. The antibodies used in this study are listed in Table S3.

### TUNEL assay

Apoptotic cells in paraffin embedded tissue sections were detected using a one-step TUNEL In Situ Apoptosis Kit (Elabscience, #E-CK-A321, China) in accordance with the manufacturer’s instructions. Briefly, tissue sections were deparaffinized, rehydrated, and permeabilized, followed by incubation with the TUNEL reaction mixture for in situ labeling of DNA strand breaks. After washing, nuclei were counterstained with DAPI, and coverslips were mounted with antifade mounting medium. TUNEL positive cells were visualized under a fluorescence microscope.

### Immunofluorescence (IF) staining

IF staining was performed using the Universal IF Toolkit (Abbkine, #KTD107, China) according to the manufacturer’s instructions. Cells were climbed, fixed, permeabilized, and blocked, followed by overnight incubation with the primary antibody and subsequent incubation with the corresponding secondary antibody for 1 h at room temperature. The slides were mounted with antifade mounting medium containing DAPI (Beyotime, #P0131, China).

For multiplex fluorescent immunohistochemical staining, all procedures were performed in accordance with the manufacturer’s protocol for the multiplex immunofluorescence staining kit (Absin, #abs50012, China). Tissue sections were first deparaffinized in xylene and then rehydrated through a graded ethanol series to prepare for subsequent multiplex staining. After antigen retrieval by microwave heating, treatment with 3% hydrogen peroxide, and blocking, the sections were sequentially incubated with primary antibodies and HRP conjugated secondary antibodies, followed by fluorophore deposition for signal amplification. Each staining cycle consisted of microwave treatment, blocking, and antibody incubation. Upon completion of all staining cycles, nuclei were counterstained with DAPI, and the sections were mounted using an antifade mounting medium. All slides were imaged using the Pannoramic MIDI Ⅱ system (3DHISTECH, Hungary). Detailed information on the antibodies used is provided in Table S3.

### RIP-qPCR assay

The acRIP assay was performed using the EpiTM ac4C Immunoprecipitation Kit (Epibiotek, #R1815, China) in accordance with the manufacturer’s instructions. In brief, 100 µg of total RNA was randomly fragmented into 100–200 bp fragments and incubated with 5 µg of anti-ac4C antibody (or IgG control) and magnetic beads. Following immunoprecipitation, ac4C modified RNAs were purified and eluted with nuclease free water. Enriched ac4C modified mRNAs were subsequently detected by qRT-PCR. The primers for acRIP-qPCR are listed in Table S2. For the NAT10 RIP assay, the anti-ac4C antibody was replaced with an anti-NAT10 antibody, while all other procedures remained unchanged.

### ChIP-qPCR assay

Chromatin immunoprecipitation (ChIP) was performed using a ChIP assay kit (Beyotime, #P2080S, China) according to the manufacturer’s protocol. Briefly, cells were fixed with 1% formaldehyde for 10 min, and the reaction was quenched with glycine for 5 min at room temperature. Cells were washed, collected, and resuspended in lysis buffer. The lysates were subjected to ultrasonication to shear genomic DNA into fragments, followed by incubation with anti-p65 antibodies for immunoprecipitation. Immunoprecipitated DNA was purified using a DNA purification kit (Beyotime, #D0033, China) and analyzed by qRT-PCR. The primers for ChIP-qPCR are listed in Table S2.

### Dual luciferase reporter gene assay

The dual luciferase reporter assay was performed using an assay kit (Abbkine, #KTA8010, China) in accordance with the manufacturer’s protocol. Cells were lysed and centrifuged, and the supernatant was collected. The lysate was transferred to a 96 well black plate. Firefly luciferase substrate was added, mixed, and immediately measured using a multifunctional microplate reader (Tristar^2^ LB 942, Germany). Renilla luciferase substrate was then added, mixed, and measured. Firefly luciferase activity was normalized to Renilla luciferase activity.

### Animal experiments

All animal experiments were approved by the Medical Experimental Animal Care Committee of the Second Affiliated Hospital of Harbin Medical University (Approval No. SYDW2025-101) and were conducted in accordance with the National Institutes of Health guidelines for animal care and use. Female BALB/c mice and BALB/c nude mice, aged 6–8 weeks, were obtained from Liaoning Changsheng Biotechnology Co., Ltd. (China). All animals were housed under specific pathogen free (SPF) conditions in the Medical Experimental Animal Center of the Second Affiliated Hospital of Harbin Medical University. Approximately 5 × 10^4^ 4T1 cells in 50 µL of serum free medium mixed with 50 µL of Matrigel (Corning, #356234, USA) were injected subcutaneously into the axillary region of mice. The HDAC4 inhibitor LMK-235 (MedChemExpress, #HY18998, China) was administered intraperitoneally at doses of 5 mg/kg or 10 mg/kg body weight every three days. The PD-L1 inhibitor (Bioxcell, #751220D1B, USA) was administered intraperitoneally at a dose of 75 µg every four days. In the CD8⁺ T cell depletion assay, mice were administered anti-Mouse CD8α antibody (Clone 2.43) (MedChemExpress, #HY-P990790, China) intraperitoneally at a dose of 300 µg daily for three consecutive days. Single cell suspensions prepared from blood were subsequently analyzed by flow cytometry to validate the extent of CD8⁺ T cell depletion. Single cell suspensions prepared from blood were isolated by using lymphocyte separation medium (Solarbio, #P8620, China). Tumor volume was measured every three days with a digital caliper and calculated as ½ × (length × width²). At the endpoint, mice were euthanized, and tumors were excised and fixed in 4% paraformaldehyde for subsequent analysis.

### Enzyme linked immunosorbent assay (ELISA)

The concentrations of Granzyme B (GzmB) and IFN-γ in mouse serum were measured with corresponding ELISA kits (Jonlnbio, JL11913 and JLW10967, China) per the manufacturer’s protocol. The procedure involved sequential incubation with samples, biotinylated detection antibody, and streptavidin-HRP, each followed by washing, before final incubation with TMB substrate, reaction termination, and absorbance measurement at 450 nm.

### acRIP-Sequencing (acRIP-seq)

The acRIP-seq experiment and subsequent data analysis were conducted by Epibiotek Co., Ltd. (Guangzhou, China). Total RNA was extracted from cells, and RNA integrity was assessed. The RNA was fragmented into 100–200 nt oligonucleotides. A small fraction was preserved as input, and the remainder was used for immunoprecipitation with an anti-ac4C antibody. ac4C modified RNAs were captured using Dynabeads, and immunoprecipitated RNA was purified. RNA-seq libraries were prepared from both input RNA and ac4C enriched RNA and sequenced on the Illumina platform. Sequencing data were aligned to the human genome using HISAT2, and ac4C peaks were identified using ExomePeak. Peaks with fold change > 2.0 and *P* < 0.05 were selected for further analysis.

### RNA-Sequencing (RNA-seq)

RNA-seq was performed by Majorbio Biotechnology Co., Ltd. (Shanghai, China) with subsequent data analysis. Total RNA was extracted, and its quality, concentration, and integrity were assessed by spectrophotometry and gel electrophoresis. Small RNA libraries were prepared using the Illumina TruSeq Small RNA Kit, and strand-specific libraries were constructed with the Illumina TruSeq RNA Sample Preparation Kit. Sequencing was performed on the Illumina NovaSeq 6000 or HiSeq X Ten platform according to the manufacturer’s instructions.

### Statistical analyses

Statistical analyses were conducted using SPSS or GraphPad Prism software. Data are presented as mean ± standard deviation (SD) from at least three independent experiments. Comparisons between two groups were performed using the Student’s t-test, and multiple group comparisons were performed using one-way ANOVA. Spearman’s correlation was applied for correlation analyses. Survival analysis was conducted using Kaplan-Meier curves with log-rank tests. Gene expression and immune related data from The Cancer Genome Atlas (TCGA) were analyzed using TIMER 2.0 (https://timer.compgenomics.org/, Seattle, WA, USA). Statistical significance was defined as *P < 0.05, **P < 0.01, ***P < 0.001, and ****P < 0.0001.

## Results

### NAT10 mediated ac4C modification promotes breast cancer progression

**Fig. 1:**
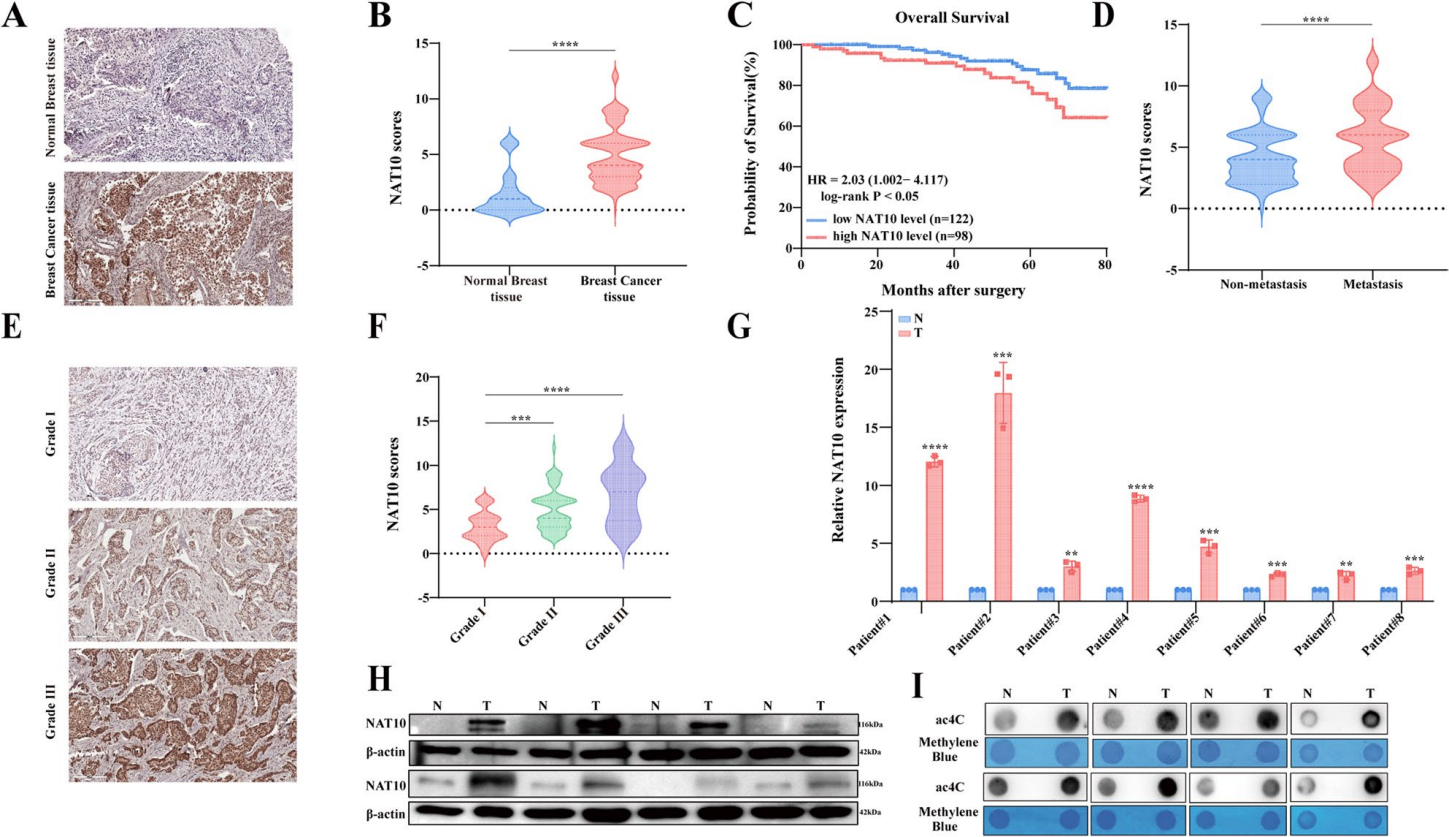
NAT10 mediated ac4C modification promotes breast cancer progression.** A**,** B** Representative IHC staining of NAT10 in normal breast tissues (*n* = 24) and breast cancer tissues (*n* = 220) (scale bar, 200 μm), with quantification of staining intensity shown in **B**. **C** Kaplan–Meier survival analysis of breast cancer patients stratified by NAT10 expression levels (low, *n* = 122; high, *n* = 98; log-rank test, *P* < 0.05). **D** NAT10 expression in breast cancer tissues with or without lymph node metastasis (metastasis, *n* = 112; non-metastasis, *n* = 108). **E**,** F** Representative IHC staining of NAT10 in breast cancer tissues of different pathological grades (Grade I, *n* = 21; Grade II, *n* = 185; Grade III, *n* = 14) (scale bar, 200 μm), with statistical analysis shown in **F**. **G-I** NAT10 expression in 8 paired breast cancer and adjacent normal tissues was analyzed by qRT-PCR (**G**), Western blot (**H**), and dot blot **I**. All data are presented as mean ± SD. * *P* < 0.05, ** *P* < 0.01, *** *P* < 0.001, **** *P* < 0.0001

To explore the role of NAT10 in breast cancer, we first examined its expression profile across cancers using the TIMER database. NAT10 mRNA expression was found to be significantly elevated in breast cancer tissues compared with normal breast tissues (Fig. S1A). To further investigate the involvement of NAT10 in breast cancer occurrence and progression, we analyzed clinical samples, including 220 breast cancer tissues and 24 normal breast tissues, by IHC. The staining results demonstrated that NAT10 expression was higher in breast cancer tissues than in normal tissues (Fig. 1A-B). Survival analysis revealed that high NAT10 expression was associated with poor prognosis in breast cancer patients (Fig. 1C). Correlation analysis between IHC scores and clinical outcomes indicated that elevated NAT10 expression was correlated with breast cancer metastasis (Fig. 1D). Furthermore, NAT10 expression was significantly increased in patients with pathological grade III compared to those with grades I and II (Fig. 1E-F). In addition, compared with adjacent normal breast tissues, NAT10 mRNA and protein levels were markedly upregulated in breast cancer tissues, as confirmed by analysis of eight randomly selected pairs of fresh surgical samples (Fig. 1G-H). Notably, consistent with NAT10 expression, ac4C levels were also increased in breast cancer tissues (Fig. 1I). These findings suggest that NAT10 mediated ac4C modification serves as a valuable biomarker associated with breast cancer progression.

### NAT10 promotes breast cancer proliferation *in vivo* and *in vitro*

**Fig. 2:**
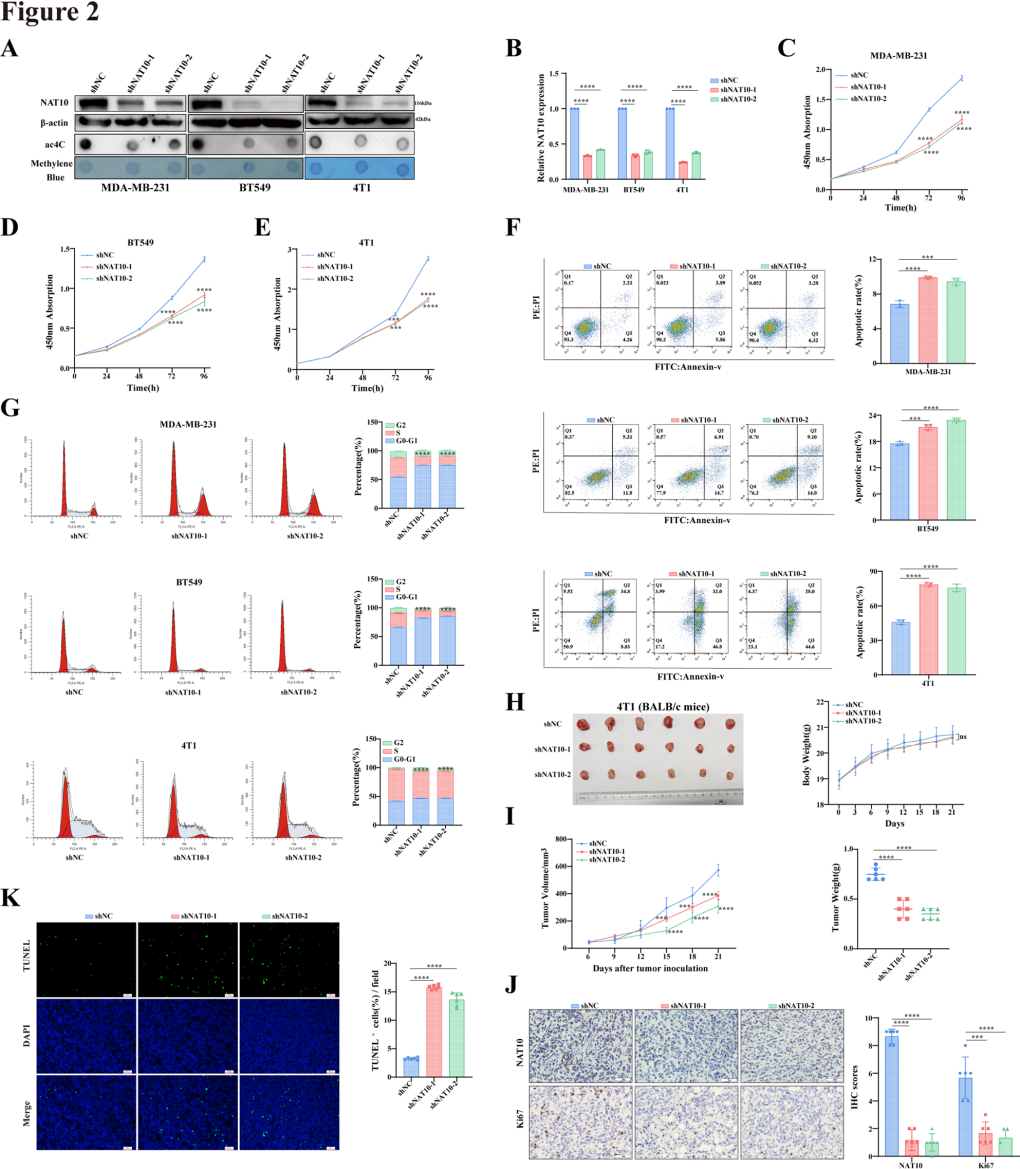
Knockdown of NAT10 inhibits breast cancer proliferation in vitro and in vivo. **A, B** NAT10 expression after knockdown was analyzed by Western blot (A), dot blot (A), and qRT-PCR (B). **C-E** Cell proliferation was assessed by CCK-8 assay in NAT10 knockdown cells. **F, G** Apoptosis rate (LR + UR) (F) and cell cycle distribution (G) were analyzed by flow cytometry in NAT10 knockdown cells. **H, I** Tumor growth in shNC and shNAT10 groups (*n* = 6) was shown, including representative tumor images (H), body weight curves (H), tumor growth curves (I), and tumor weights (I). **J** Representative IHC staining of NAT10 and Ki67 in tumor tissues from shNC and shNAT10 groups (scale bars, 50 µm), with quantification of staining intensity (*n* = 6). **K** Representative TUNEL staining of tumor tissues from shNC and shNAT10 groups (scale bars, 100 px), with quantification of positive cells (*n* = 6). All data are presented as mean ± SD. * *P* < 0.05, ** *P* < 0.01, *** *P* < 0.001, **** *P* < 0.0001

**Fig. 3:**
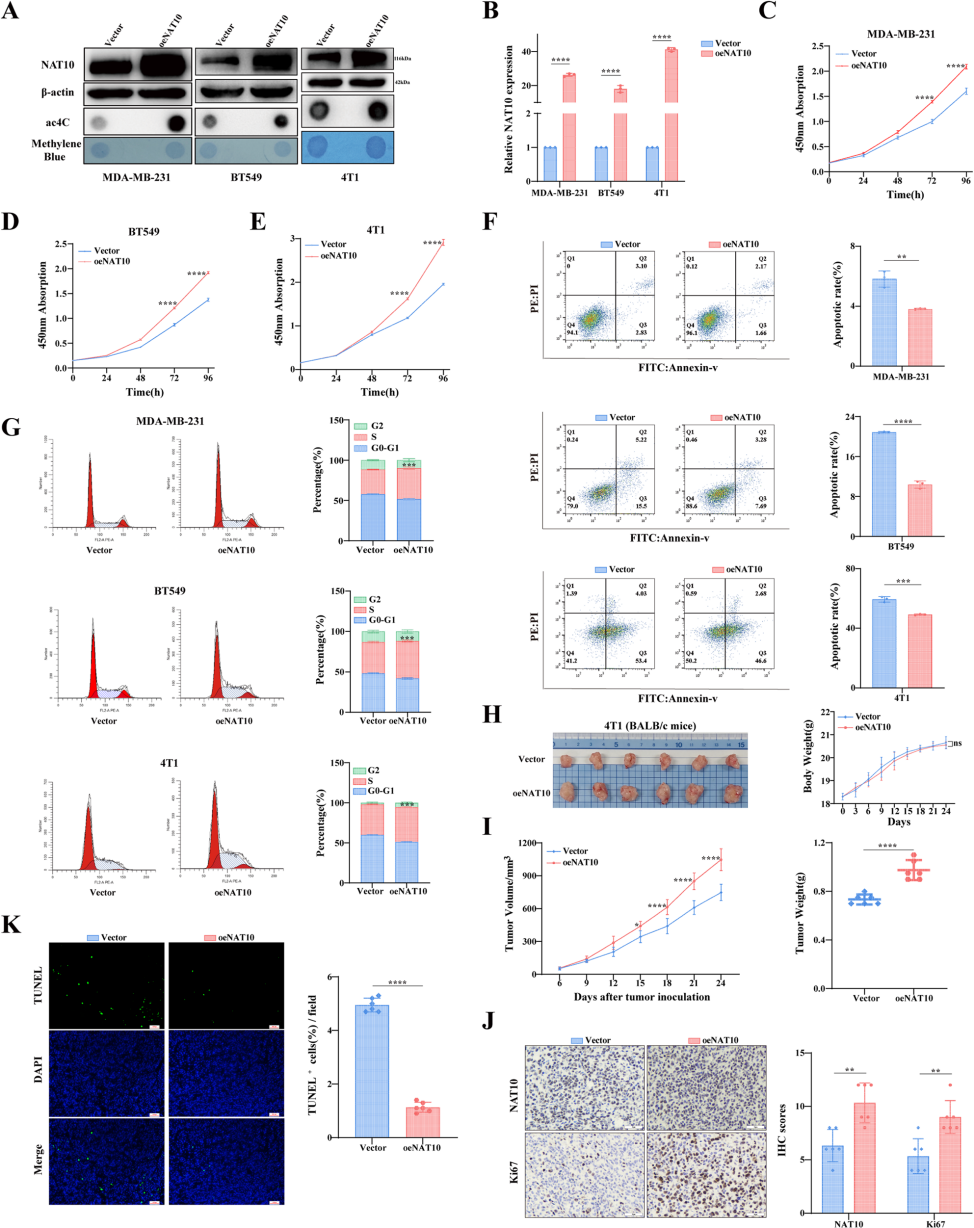
Overexpression of NAT10 promotes breast cancer cell proliferation *in vitro* and *in vivo*. **A**,** B** NAT10 expression after overexpression was analyzed by Western blot (**A**), dot blot (**A**), and qRT-PCR (**B)**. **C-E** Cell proliferation was assessed by CCK-8 assay in NAT10 overexpressing cells. **F**,** G** Apoptosis rate (LR + UR) (**F**) and cell cycle distribution (**G**) were analyzed by flow cytometry in NAT10 overexpressing cells. **H**,** I** Tumor growth in vector and oeNAT10 groups (*n* = 6) is shown, including representative tumor images (**H**), body weight curves (**H**), tumor growth curves (**I**), and tumor weights (**I)**. **J** Representative IHC staining of NAT10 and Ki67 in tumor tissues from vector and oeNAT10 groups (scale bars, 50 μm), with quantification of staining intensity (*n* = 6). **K** Representative TUNEL staining of tumor tissues from vector control and oeNAT10 groups (scale bars, 100 px), with quantification of positive cells (*n* = 6). All data are presented as mean ± SD. * *P* < 0.05, ** *P* < 0.01, *** *P* < 0.001, **** *P* < 0.0001

To investigate the effect of NAT10 on breast cancer growth, stable NAT10 knockdown and overexpression breast cancer cell lines were generated using lentiviral vectors, and expression levels were quantified and validated by Western blot and qRT-PCR (Figs. 2A-B and 3A-B). To exclude potential off-target effects of the shRNA, we generated shRNA resistant constructs by introducing silent mutations into the wild-type NAT10 sequence (NAT10^WT − res^) and into the catalytically inactive G641E mutant (NAT10^G641E − res^). Western blot analysis showed that the introduced NAT10^WT − res^ and NAT10^G641E − res^ constructs were stably expressed and fully restored NAT10 protein levels that had been reduced by shRNA mediated knockdown (Fig. S1B). Quantification of ac4C levels confirmed that mRNA ac4C modification varied with NAT10 expression (Figs. 2A-B and 3A-B). CCK-8 assays demonstrated that NAT10 enhanced cell proliferation (Figs. 2C-E and 3C-E). Flow cytometric analysis revealed that NAT10 knockdown increased apoptosis and inhibited the G1-to-S phase transition, whereas NAT10 overexpression produced the opposite effects (Figs. 2F-G and 3F-G). In tumor bearing mice injected with 4T1 cells, both tumor weight and tumor volume analyses indicated that NAT10 knockdown suppressed tumor growth, while NAT10 overexpression promoted tumor growth (Figs. 2H-I and 3H-I). No significant differences in body weight were observed among the groups (Figs. 2H-I and 3H-I). IHC analysis showed significantly decreased Ki67 expression in the shNAT10 group compared with the control group, while Ki67 expression was significantly increased in the oeNAT10 group (Figs. 2J and 3J). TUNEL staining further demonstrated that the proportion of TUNEL positive cells increased in the shNAT10 group but decreased in the oeNAT10 group (Figs. 2K and 3K). Collectively, these findings demonstrate that NAT10 plays a critical role in promoting breast cancer cell proliferation, suggesting that it may serve as a potential therapeutic target.

### NAT10 stimulates HDAC4 expression via ac4C modification

**Fig. 4:**
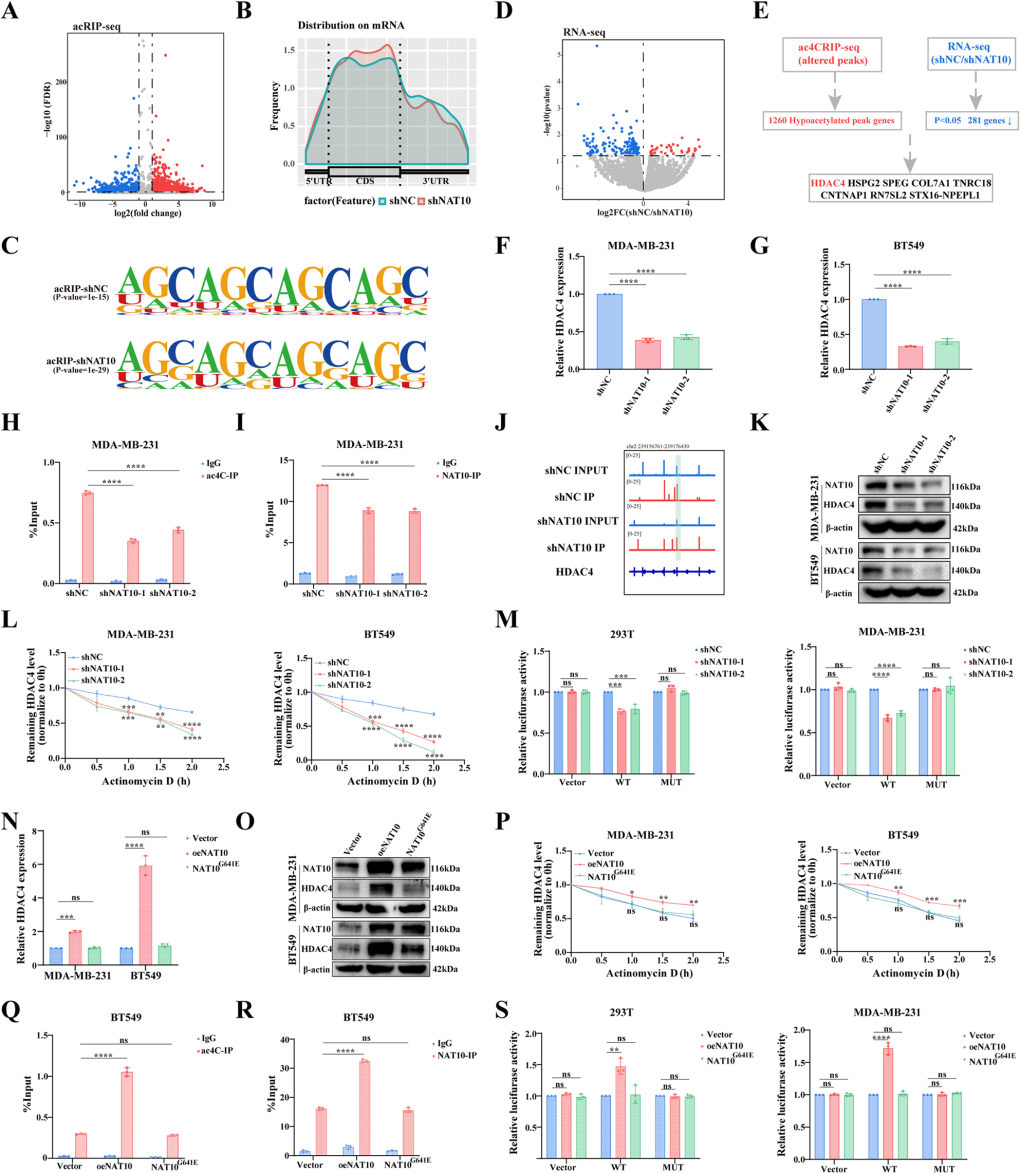
NAT10 stimulates HDAC4 expression via ac4C modification. **A** Volcano plot of differentially expressed ac4C acetylation peaks (*P* < 0.05) identified by acRIP-seq in NAT10 knockdown cells. **B** Distribution of ac4C peaks across mRNA regions in breast cancer cells. **C** Sequence logo of the enriched motif within ac4C peaks identified by HOMER. **D** Volcano plot of differentially expressed mRNAs (*P* < 0.05) identified by RNA-seq in NAT10 knockdown cells. **E** Integrative analysis of acRIP-seq and RNA-seq data to identify potential downstream targets of NAT10. **F**,** G** Relative HDAC4 mRNA expression measured by qRT-PCR after NAT10 knockdown. **H**,** I** acRIP-qPCR analysis of ac4C modification (**H**) and RIP-qPCR analysis of NAT10 binding on HDAC4 mRNA (**I**) after NAT10 knockdown. **J** Genome browser view of ac4C peaks on HDAC4 mRNA from acRIP-seq. **K** Relative HDAC4 protein levels measured by Western blot after NAT10 knockdown. **L** qRT-PCR analysis of HDAC4 mRNA stability after actinomycin D treatment in NAT10 knockdown cells. **M** Luciferase activity of the reporter constructs containing the wild-type or ac4C site mutated sequence in NAT10 knockdown cells. **N**,** O** HDAC4 expression assessed by qRT-PCR (**N**) and Western blot (**O**) after transfection with oeNAT10 or NAT10^G641E^mutant plasmid. **P** qRT-PCR analysis of HDAC4 mRNA stability after actinomycin D treatment in cells transfected with oeNAT10 or NAT10^G641E^. **Q**,** R** acRIP-qPCR analysis of ac4C modification (**Q**) and RIP-qPCR analysis of NAT10 binding on HDAC4 mRNA (**R**) after transfection with oeNAT10 or NAT10^G641E^. **S** Luciferase activity of the reporter constructs containing the wild-type or ac4C site mutated sequence after transfection with oeNAT10 or NAT10^G641E^. All data are presented as mean ± SD. * *P* < 0.05, ** *P* < 0.01, *** *P* < 0.001, **** *P* < 0.0001

To clarify the underlying mechanism through which NAT10 drives breast cancer progression, acRIP-seq was conducted in stable NAT10 knockdown MDA-MB-231 cells and integrated with RNA-seq analysis to screen for potential target genes with concurrent downregulation of both mRNA expression and ac4C modification. acRIP-seq detected 3402 differential ac4C peaks, among which 1583 were downregulated (Fig. 4A). Distribution analysis revealed that most mRNA ac4C peaks were located in the coding sequence (CDS) and the 3′ untranslated region (3′ UTR) (Fig. 4B; Fig. S2A), consistent with previous reports [9]. Motif analysis further showed that most ac4C sites were located within the classical consensus “CXXCXXCXX” motif (Fig. 4C). Gene Ontology-biological process (GO-BP) analysis of the differential genes revealed enrichment in RNA splicing, mRNA processing, and nuclear transport (Fig. S2B). Kyoto Encyclopedia of Genes and Genomes (KEGG) pathway analysis indicated enrichment in protein processing in the endoplasmic reticulum, RNA transport, and spliceosome pathways (Fig. S2C). RNA-seq identified 342 differentially expressed genes (DEGs), of which 281 were downregulated (Fig. 4D). Cross analysis of acRIP-seq and RNA-seq identified eight genes (Table S4) with reduced ac4C modification and decreased mRNA expression following NAT10 knockdown (Fig. 4E). Among these candidate genes, HDAC4 showed the most pronounced reduction in both ac4C modification and mRNA abundance following NAT10 knockdown (Fig. 4F-I, Fig. S2D-F). Consequently, HDAC4 was selected as the primary target for subsequent investigation.

Further analysis of acRIP-seq revealed a marked reduction in ac4C enrichment in HDAC4 (Fig. 4J). Consistently, Western blot analysis demonstrated that HDAC4 protein expression was reduced in NAT10 knockdown cells (Fig. 4K). mRNA stability assays using actinomycin D further revealed that NAT10 knockdown significantly shortened the half-life of HDAC4 mRNA, thereby promoting its degradation (Fig. 4L). To validate the functional importance of ac4C modification, dual luciferase reporter constructs were generated containing wild-type (WT) or ac4C site mutant (MUT) HDAC4 transcript sequences. NAT10 knockdown significantly decreased luciferase activity of the WT reporter, but had no effect on the MUT reporter (Fig. 4M). Previous studies have shown that mutations in conserved glycine residues of NAT10, particularly substitution of glycine with glutamic acid (G641E), abolish its acetyltransferase activity [20, 21]. Building on these findings, we next sought to explore the relationship between HDAC4 expression and NAT10 catalyzed acetylation. To this end, either wild-type NAT10 (oeNAT10) or the catalytically inactive mutant (NAT10^G641E^) was transfected into MDA-MB-231 and BT549 cells. Compared with oeNAT10, the NAT10^G641E^ mutant had no significant effect on HDAC4 mRNA expression (Fig. 4N), protein expression (Fig. 4O), and also lost the ability to stabilize HDAC4 (Fig. 4P). acRIP-qPCR or RIP-qPCR analysis demonstrated that the NAT10^G641E^ mutant was not enriched in ac4C modification or HDAC4 mRNA (Fig. 4Q-R). Consistent results were obtained from the luciferase reporter assay (Fig. 4S). Furthermore, when endogenous NAT10 was reduced, overexpression of NAT10, but not the NAT10^G641E^ mutant, significantly enhanced HDAC4 expression at both the mRNA and protein levels (Fig. S2E-F). ac4C dot blot analysis revealed that oeNAT10 restored the overall ac4C modification level, whereas the NAT10^G641E^ mutant produced no significant change (Fig. S2E-F). These findings indicated that NAT10 enhanced the stability of HDAC4 mRNA in an ac4C dependent manner. Taken together, our results demonstrated that NAT10 mediated ac4C modification is essential for maintaining the stability of HDAC4 mRNA in breast cancer cells.

### HDAC4 interacts with NAT10 to promote breast cancer cell proliferation

**Fig. 5:**
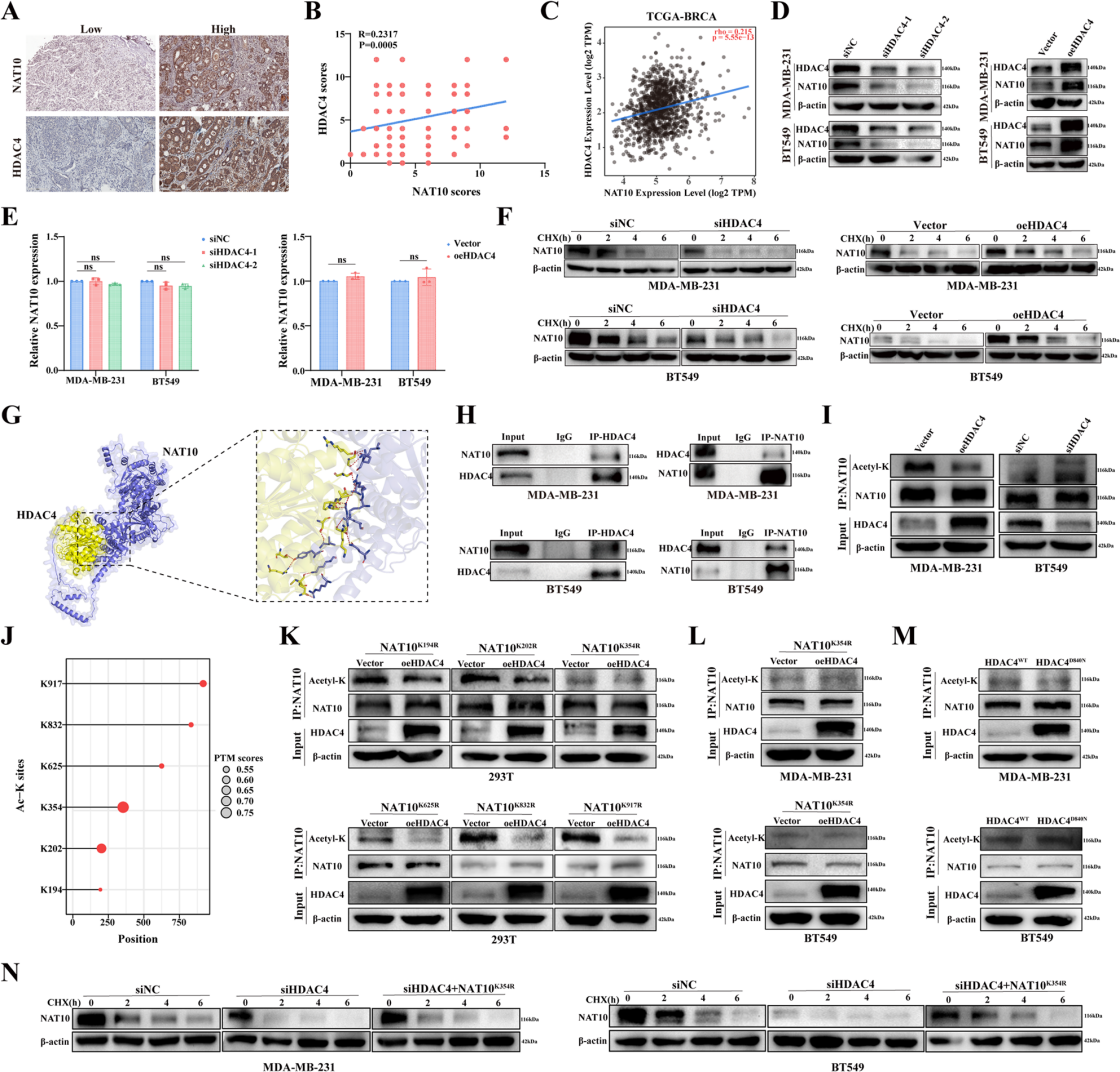
HDAC4 acts as a deacetylase to regulate NAT10 stability. **A** Representative IHC images and quantification of NAT10 and HDAC4 expression in breast cancer tissues (*n* = 220; scale bar, 200 µm). **B**, **C** Correlation between NAT10 and HDAC4 expression levels in breast cancer tissues, as analyzed by IHC (**B**) and in the TCGA-BRCA RNA-seq dataset (**C)**. **D**, **E** Relative NAT10 expression was detected by Western blot (**D**) and qRT-PCR (**E**) after HDAC4 knockdown or overexpression. **F** Western blot analysis of NAT10 protein stability after cycloheximide treatment in cells with HDAC4 knockdown or overexpression. **G** Molecular docking model of the NAT10 (blue) and HDAC4 (yellow) interaction, with an enlarged view highlighting predicted hydrogen bonds. **H** Co-IP followed by Western blot analysis was used to detect the interaction between endogenous HDAC4 and NAT10. **I** Co-IP followed by Western blot analysis assessed NAT10 acetylation levels after HDAC4 knockdown or overexpression. **J** Prediction of potential deacetylation sites on NAT10 using MusiteDeep. **K** The predicted acetylation sites on NAT10 were individually mutated (K→R), and the effects of HDAC4 on the acetylation levels of the six NAT10 mutants were examined in 293T cells. **L** Detection of NAT10 acetylation in cells expressing NAT10^K354R^ following transfection with vector or HDAC4. **M** Detection of NAT10 acetylation in cells transfected with wild-type or catalytically inactive (D840N) HDAC4. **N** Western blot analysis of NAT10 protein stability following cycloheximide treatment in cells transfected with siNC, siHDAC4, or siHDAC4 plus NAT10^K354R^. All data are presented as mean ± SD. * *P* < 0.05, ** *P* < 0.01, *** *P* < 0.001, **** *P* < 0.0001

To further examine the relationship between NAT10 and HDAC4 expression in breast cancer, we assessed their levels in clinical samples. Consistently, a positive correlation was observed between NAT10 and HDAC4 protein expression (Fig. 5A-B). Analysis of the TCGA-BRCA database also demonstrated a positive correlation between NAT10 and HDAC4 expression (Fig. 5C). Given that HDAC4 regulates not only histone acetylation but also post-translational modifications of non-histone substrates, thereby influencing their stability or activity [22–25], we hypothesized that HDAC4 might reciprocally regulate NAT10 stability through deacetylation. To test this hypothesis, HDAC4 expression was modulated in breast cancer cells, and its impact on NAT10 levels was assessed. Interestingly, HDAC4 knockdown reduced NAT10 protein levels, whereas HDAC4 overexpression increased them (Fig. 5D); by contrast, no significant changes were seen in NAT10 mRNA levels according to qRT-PCR analysis (Fig. 5E; Fig. S3A), indicating that HDAC4 regulates NAT10 at the post-translational level. To determine whether HDAC4 directly affects NAT10 stability, cycloheximide chase experiments were performed. These experiments demonstrated that HDAC4 knockdown shortened the half-life of NAT10 protein, whereas HDAC4 overexpression delayed its degradation (Fig. 5F), supporting the conclusion that HDAC4 stabilizes NAT10. To explore the potential molecular basis of this regulation, GRAMM based structural predictions were performed, suggesting a direct interaction between HDAC4 and NAT10 (Fig. 5G). Co-IP assays further confirmed the interaction between endogenous HDAC4 and NAT10 (Fig. 5H). Finally, to assess the functional consequence of this interaction on post-translational modification, NAT10 acetylation levels were analyzed. Knockdown of HDAC4 increased NAT10 acetylation, whereas HDAC4 overexpression reduced it (Fig. 5I). These findings indicated that HDAC4 regulates the acetylation level of NAT10, thereby affecting its protein stability.

To identify the specific lysine residues on NAT10 regulated by HDAC4, we first used MusiteDeep (https://www.musite.net/) to predict lysines with acetylation potential. Six candidate residues were identified: K194, K202, K354, K625, K832, and K917 (Fig. 5J). Each lysine was then individually mutated to arginine(K→R) to prevent acetylation, and the impact of HDAC4 on these mutants was examined in 293T cells. Among all mutants tested, the K354R substitution uniquely abolished the regulatory effect of HDAC4, indicating that K354 is the critical site through which HDAC4 modulates NAT10 (Fig. 5K). Consistently, analysis in breast cancer cells confirmed that HDAC4 regulates the acetylation level of NAT10 specifically at the K354 residue (Fig. 5L). We further assessed NAT10 acetylation in the context of the catalytically inactive HDAC4^D840N^ mutant [26]. As shown in Fig. 5M, the HDAC4^D840N^ mutant failed to alter NAT10 acetylation, demonstrating that HDAC4’s deacetylase activity is required for controlling the acetylation state of NAT10. We further employed cycloheximide chase assays to assess protein stability. The results showed that under HDAC4 knockdown conditions, preventing acetylation at the K354 site through the K354R mutation maintained the stability of the NAT10 protein (Fig. 5N). These findings further demonstrate that K354 is a critical acetylation acceptor site. Overall, HDAC4 was regulated by NAT10 as a downstream target gene and, in turn, acted as a deacetylase to regulate NAT10 stability.

**Fig. 6:**
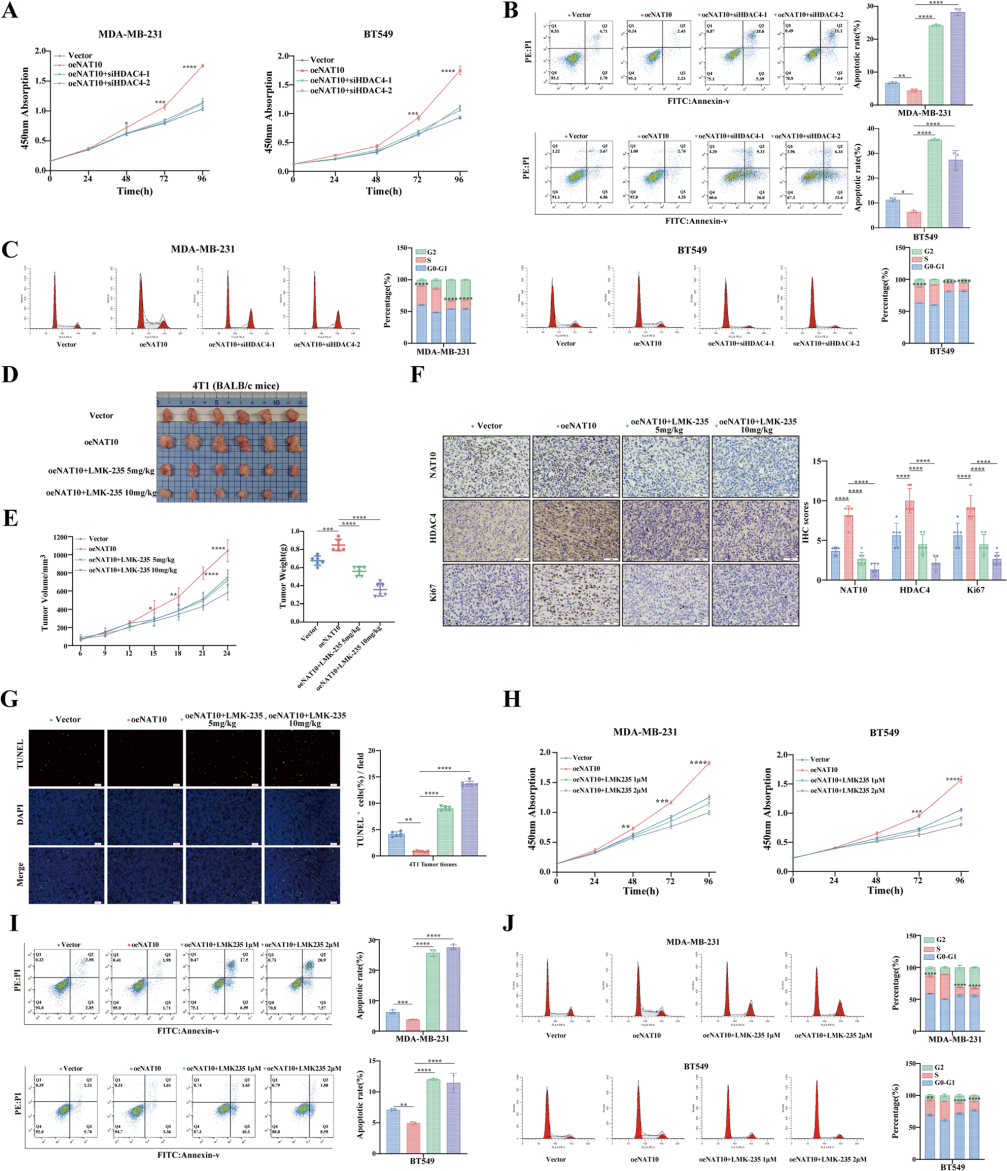
The proliferative function of NAT10 in breast cancer depends on HDAC4.** A** CCK-8 assays of cells transfected with vector, oeNAT10, oeNAT10 plus siHDAC4-1, or oeNAT10 plus siHDAC4-2. **B** Flow cytometry analysis of apoptotic rates (LR + UR) in cells transfected with the indicated constructs. **C** Flow cytometry analysis of cell cycle distribution in cells transfected with the indicated constructs. **D** Representative images of tumors in mice treated with vector, oeNAT10, oeNAT10 plus LMK235 (5 mg/kg), or oeNAT10 plus LMK235 (10 mg/kg) (*n* = 6). **E** Tumor growth curves and tumor weight analysis in tumors from different groups (*n* = 6). **F** Representative IHC images of NAT10, HDAC4, and Ki67 in tumor tissues from different treatment groups, with quantification of staining intensity (*n* = 6; scale bars, 50 μm). **G** Representative TUNEL staining of tumor tissues from different treatment groups, with quantification of staining intensity (*n* = 6; scale bars, 100 px). **H-J** CCK-8 assays (**H**) and flow cytometry analyses of apoptotic rates (**I**) and cell cycle distribution (**J**) in cells transfected with vector, oeNAT10, oeNAT10 plus LMK235 (1 µM), or oeNAT10 plus LMK235 (2 µM). All data are presented as mean ± SD. * *P* < 0.05, ** *P* < 0.01, *** *P* < 0.001, **** *P* < 0.0001

To investigate whether HDAC4 is required for the oncogenic effects of NAT10, we evaluated cell proliferation, apoptosis, and cell cycle distribution in breast cancer cells. NAT10 overexpression induced cell proliferation was attenuated by HDAC4 knockdown (Fig. 6A). Flow cytometry analysis revealed that knockdown of HDAC4 significantly reversed the reduction of apoptotic cells and the increase of S-phase cells caused by NAT10 overexpression (Fig. 6B-C). Consistent with the in vitro results, in vivoexperiments demonstrated that the HDAC4 inhibitor LMK235 attenuated the tumorigenic effect of NAT10 overexpression (Fig. 6D-E). Moreover, the LMK235 administration did not significantly alter the body weight of the mice (Fig. S3B). H&E staining of major organs, including the heart, lung, liver, and kidney, revealed no observable changes (Fig. S3C). Myocardial fibers remained orderly, hepatic lobules were structurally intact, splenic red and white pulp were well preserved, alveolar architecture was normal, and renal glomeruli and tubules showed no pathological changes. Similar results were obtained by IHC detection of NAT10, HDAC4, Ki67 expression and TUNEL staining (Fig. 6F-G). Moreover, LMK235 markedly suppressed NAT10 induced proliferation (Fig. 6H) and largely reversed both the reduction in apoptotic cells (Fig. 6I) and the increase in S-phase cells caused by NAT10 overexpression (Fig. 6J). These findings suggested that the pro-proliferative effect of NAT10 in breast cancer is dependent on HDAC4. Next, we employed the HDAC4^D840N^ mutant to confirm whether LMK-235 exerts its effects specifically by inhibiting HDAC4. In NAT10 overexpressing cells treated with LMK235, reintroduction of wild-type HDAC4 restored the phenotypic changes induced by the drug, including enhanced cell proliferation (Fig. S3D), reduced apoptosis (Fig. S3E), and an increased proportion of S-phase cells (Fig. S3F). In contrast, the catalytically inactive HDAC4 mutant was unable to rescue any of these effects. Although LMK-235 may have additional potential targets, the inability of the D840N mutant to restore these phenotypes effectively rules out nonspecific effects. Together, these findings confirm that the biological activity of LMK-235 is indeed mediated through inhibition of HDAC4 catalytic function. Overall, our study demonstrated that the reciprocal interaction between NAT10 and HDAC4 amplifies NAT10 driven breast cancer cell proliferation and tumor progression, suggesting that targeting the NAT10-HDAC4 signaling axis may represent a potential therapeutic strategy for breast cancer.

### NAT10 promotes PD-L1 expression via the HDAC4-NF-κB pathway

**Fig. 7:**
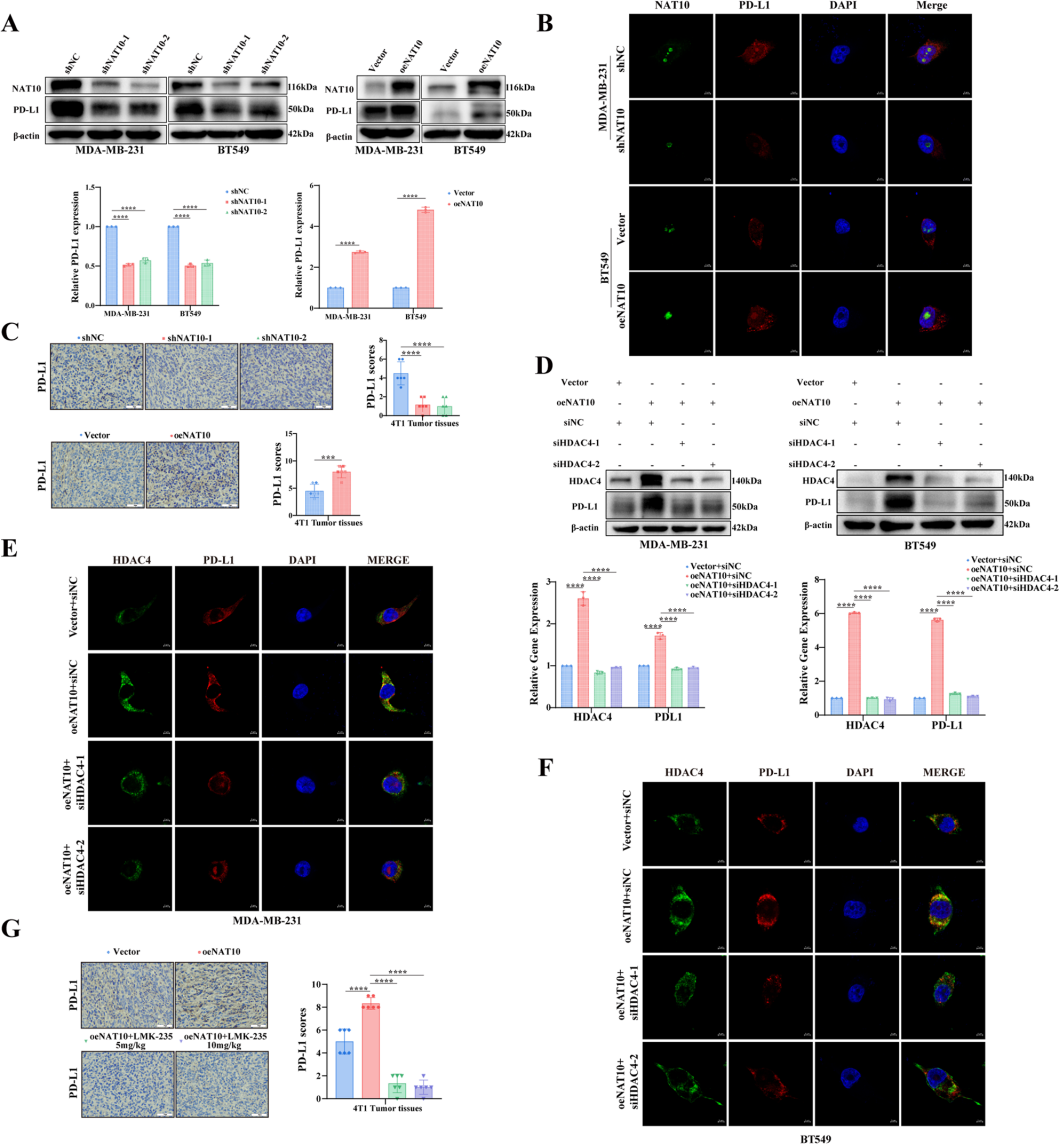
NAT10 promotes PD-L1 expression through HDAC4. **A** Relative PD-L1 expression assessed by Western blot and qRT–PCR following NAT10 knockdown or overexpression. **B** IF staining of PD-L1 in cells with NAT10 knockdown or overexpression (scale bars, 5 μm). **C** Representative IHC images of PD-L1 in tumor tissues from shNC or shNAT10 groups and vector or oeNAT10 groups, with quantification of staining intensity (*n* = 6; scale bars, 50 μm). **D-F** Relative PD-L1 expression analyzed by Western blot (**D**), qRT-PCR (**D**), and IF (**E-F**) in cells transfected with vector, oeNAT10, oeNAT10 plus siHDAC4-1, or oeNAT10 plus siHDAC4-2. **G** Representative IHC images of PD-L1 in tumor tissues from vector, oeNAT10, oeNAT10 plus LMK235 (5 mg/kg), or oeNAT10 plus LMK235 (10 mg/kg), with quantification of staining intensity (*n* = 6; scale bars, 50 μm) All data are presented as mean ± SD. * *P* < 0.05, ** *P* < 0.01, *** *P* < 0.001, **** *P* < 0.0001

To further elucidate how the NAT10-HDAC4 axis promotes breast cancer progression, we considered our previous finding that NAT10 enhances glycolysis to suppress T cell infiltration and activation in breast cancer [27]. Given the central role of PD-L1/ programmed death-1 (PD-1) signaling in tumor immune evasion [28, 29], we next investigated whether NAT10 regulates PD-L1 expression. Remarkably, NAT10 knockdown markedly reduced, whereas NAT10 overexpression increased, PD-L1 protein and mRNA levels (Fig. 7A). Consistently, IF staining revealed decreased intracellular PD-L1 expression in NAT10 knockdown cells and increased expression in NAT10 overexpressing cells (Fig. 7B). IHC analysis further confirmed these findings, showing reduced PD-L1 expression in tumors from shNAT10 groups and elevated PD-L1 expression in oeNAT10 groups compared with controls (Fig. 7C). Notably, HDAC4 knockdown significantly diminished NAT10 induced upregulation of PD-L1 at both mRNA and protein levels (Fig. 7D), and this reversal effect was consistently observed in IF (Fig. 7E-F) and IHC analyses (Fig. 7G). These results indicated that NAT10 regulates PD-L1 expression through HDAC4.

**Fig. 8:**
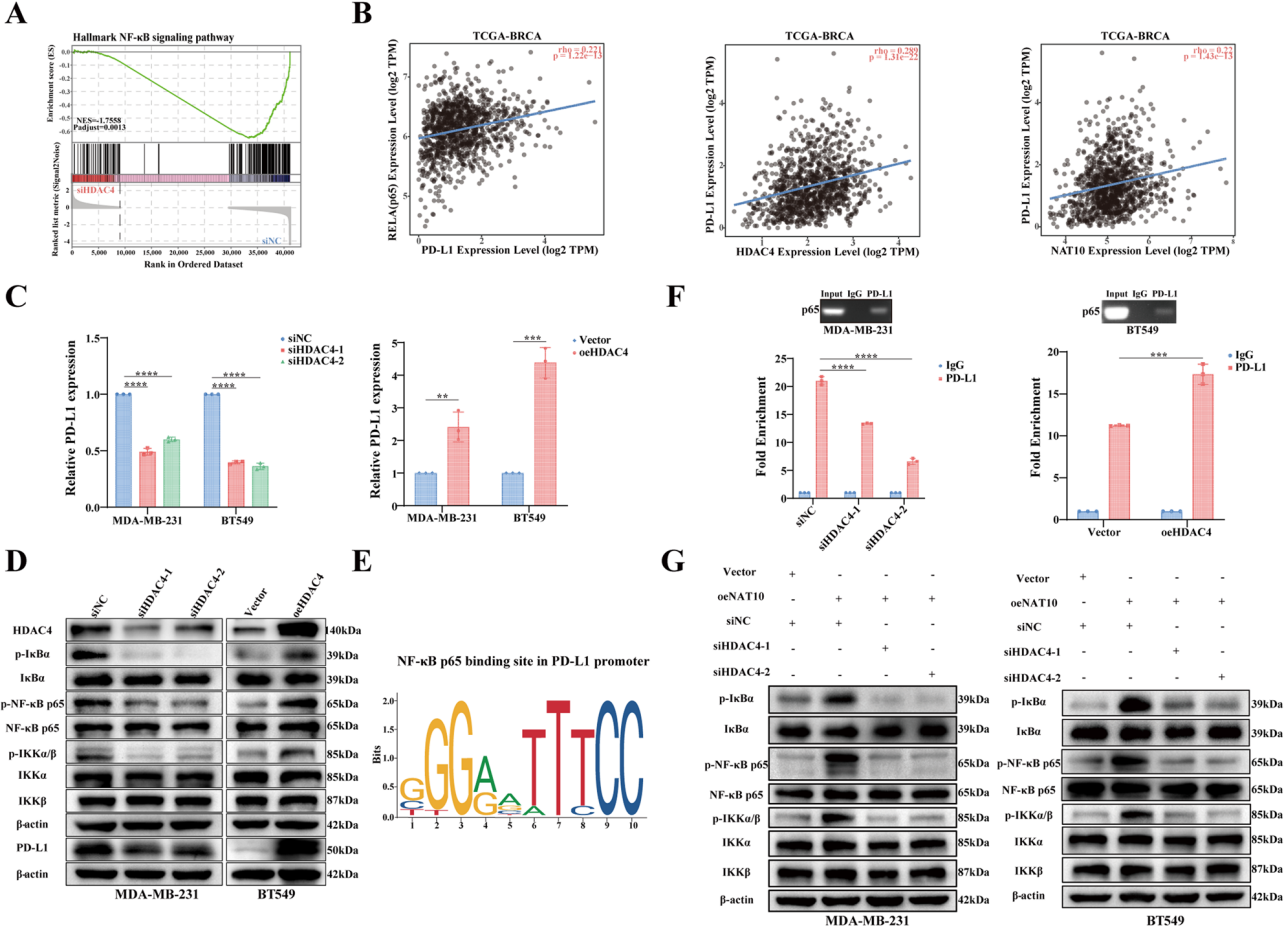
NAT10 promotes PD-L1 expression through the HDAC4–NF-κB pathway.** A** GSEA of RNA-seq (siHDAC4 vs. siNC) data showed enrichment of the NF-κB signaling pathway (NES, normalized enrichment score; *P* value by permutation test). **B** Correlation analysis of p65, NAT10, HDAC4, and PD-L1 mRNA expression in the TCGA RNA-seq dataset. **C** Relative PD-L1 expression assessed by qRT-PCR following HDAC4 knockdown or overexpression. **D **Western blot analysis of PD-L1 and NF-κB pathway proteins upon HDAC4 knockdown or overexpression. **E** Predicted p65 binding sites in the PD-L1 promoter identified using the JASPAR database. **F** ChIP-qPCR analysis of p65 enrichment at the PD-L1 promoter after HDAC4 knockdown or overexpression. **G** Western blot analysis of NF-κB pathway proteins in cells transfected with vector, oeNAT10, oeNAT10 plus siHDAC4-1, or oeNAT10 plus siHDAC4-2. All data are presented as mean ± SD. * *P* < 0.05, ** *P* < 0.01, *** *P* < 0.001, **** *P* < 0.0001

To further investigate the molecular mechanism by which the NAT10-HDAC4 axis regulates PD-L1 expression, RNA-seq followed by gene set enrichment analysis (GSEA) was performed in HDAC4 knockdown cells. We found that the NF-κB signaling pathway was significantly enriched, with a high normalized enrichment score (NES) (Fig. 8A). Given that NF-κB signaling has been reported to promote PD-L1 transcription through enhanced phosphorylation and promoter binding of p65 [30], we next examined whether HDAC4 regulates PD-L1 expression via the NF-κB axis. Analysis of the TCGA-BRCA dataset demonstrated that the expression levels of p65, NAT10, and HDAC4 were positively correlated with PD-L1 expression (Fig. 8B). Consistently, HDAC4 knockdown or overexpression resulted in decreased or increased PD-L1 mRNA expression, respectively (Fig. 8C). Furthermore, HDAC4 knockdown suppressed NF-κB signaling activity, reduced p65 phosphorylation, and consequently decreased PD-L1 protein levels, whereas HDAC4 overexpression exerted the opposite effects by further enhancing p65 phosphorylation and increasing PD-L1 expression (Fig. 8D). JASPAR analysis identified putative p65 binding sites within the PD-L1 promoter (Fig. 8E), and ChIP-qPCR assays confirmed the direct binding of p65 to the PD-L1 promoter region (Fig. 8F). Importantly, inhibition of HDAC4 in NAT10 overexpressing cells markedly attenuated NF-κB activation and reduced p65 phosphorylation, thereby diminishing NAT10 induced PD-L1 upregulation (Fig. 8G). These findings collectively demonstrated that NAT10 promotes PD-L1 expression in breast cancer cells through the HDAC4/NF-κB signaling axis. Overall, these results suggested that the NAT10-HDAC4-NF-κB pathway contributes not only to tumor cell proliferation but also to immune evasion, highlighting a potential therapeutic target in breast cancer.

### Inhibition of NAT10 enhances the efficacy of anti-PD-L1 therapy *in vivo*

**Fig. 9:**
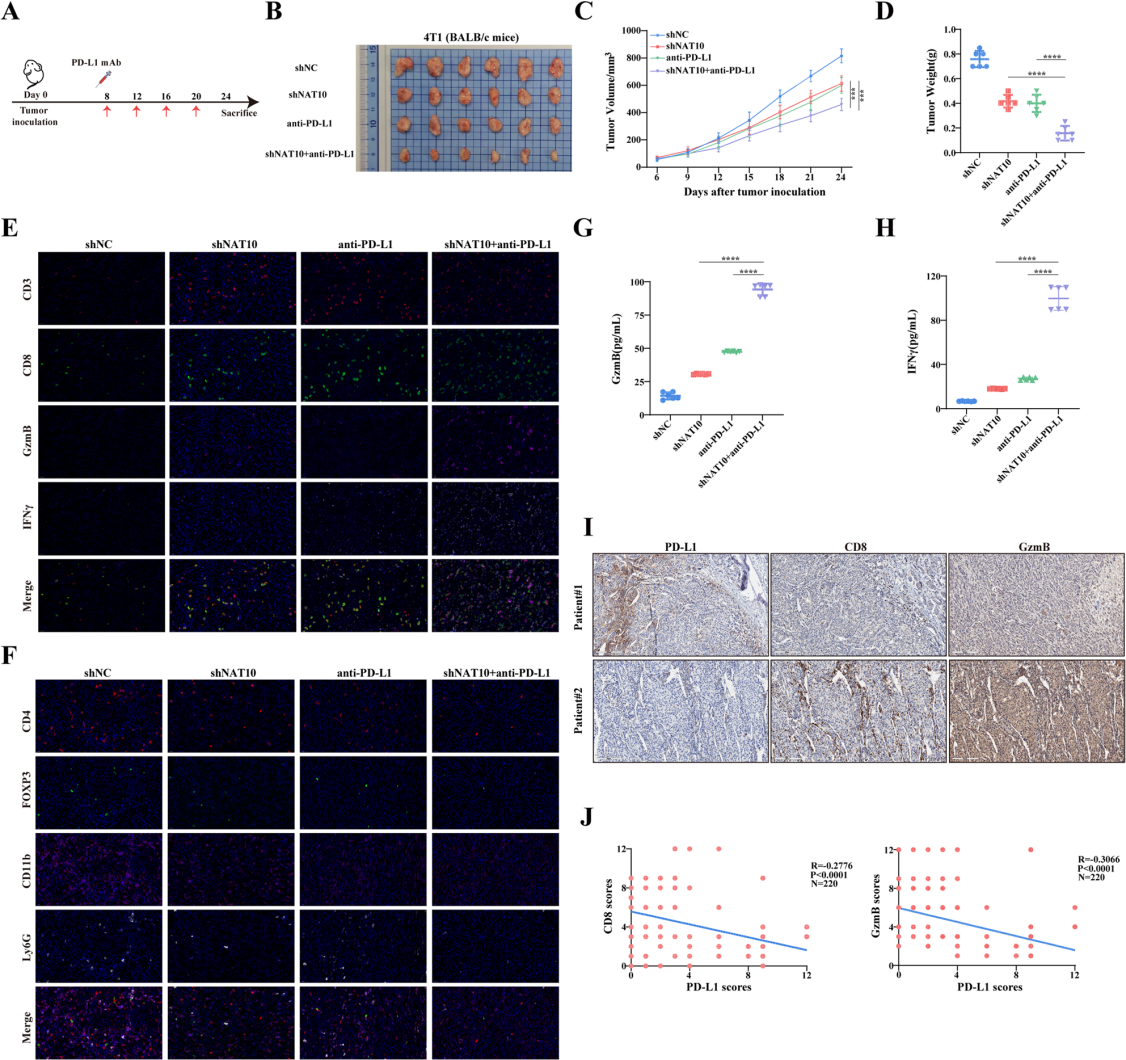
Inhibition of NAT10 enhances the efficacy of anti–PD-L1 therapy *in vivo*. **A **Schematic diagram of the tumor model and treatment schedule with anti-PD-L1 administration. **B–D** Representative images (**B**), tumor growth curves (**C**), and tumor weights (**D**) of mouse 4T1 tumors from shNC, shNAT10, anti-PD-L1, or the combination of shNAT10 and anti-PD-L1 (*n* = 6). **E-F** Representative mIF images of mouse tumor tissues showing staining for T cells (**E**), Tregs (**F**), and MDSCs (**F**) (scale bar, 20 μm). **G**-**H** ELISA analysis of GzmB **(G)** and IFN-γ** (H)** levels in the serum of mice from different groups. **I** Representative IHC staining of PD-L1, CD8, and GzmB in breast cancer tissues (*n* = 220), with statistical analysis of staining intensity (scale bar, 200 μm). **J** Correlation of PD-L1 and CD8, and PD-L1 and GzmB protein levels in breast cancer tissues (*n* = 220) assessed by IHC. All data are presented as mean ± SD. * *P* < 0.05, ** *P* < 0.01, *** *P* < 0.001, **** *P* < 0.0001

Given the role of NAT10 in modulating PD-L1 expression in breast cancer cells, in vivo experiments were conducted to evaluate whether NAT10 similarly regulates tumor growth and immune evasion in a physiological context. 4T1 cells infected with shNC or shNAT10 lentiviruses were subcutaneously injected into immunocompetent mice, followed by treatment with anti-PD-L1 antibody (Fig. 9A). Compared with the control group, tumor growth was reduced by either NAT10 knockdown or anti-PD-L1 treatment alone. At the end of the experiment, both tumor volumes and weights were lower in the shNAT10 and anti-PD-L1 groups compared with control groups (Fig. 9B-D). Notably, combined treatment further suppressed tumor growth, resulting in the greatest reduction in both tumor volume and weight (Fig. 9B-D). No significant changes in body weight were observed among mice receiving different treatment regimens (Fig. S4A). Consistently, histological examination by H&E staining revealed preserved tissue integrity of the heart, lung, liver, and kidney, with no treatment-related pathological lesions detected (Fig. S4B). These observations suggest that the treatment regimens are well tolerated in vivo, underscoring their translational potential as safe and effective therapeutic strategies. Additionally, comprehensive immune profiling of tumor tissues using multiplex immunofluorescence showed that both NAT10 knockdown and anti-PD-L1 treatment enhanced intratumoral CD8⁺ T cell infiltration and effector function (IFN-γ⁺, GzmB⁺), while reducing the abundance of regulatory T cells (Tregs) and Myeloid-derived suppressor cells (MDSCs). The combination therapy elicited the strongest immune activating response (Fig. 9E-F). Consistent with these results, ELISA analysis demonstrated substantially increased levels of GzmB and IFN-γ in mice receiving the combined shNAT10 plus anti-PD-L1 treatment, further supporting enhanced CD8⁺ T cell mediated antitumor immunity (Fig. 9G-H). In contrast, immunodeficient mice bearing shNC or shNAT10 transduced 4T1 tumors exhibited markedly different responses compared with immunocompetent hosts. In this setting, the growth inhibitory effect of NAT10 knockdown was noticeably diminished (Fig. S4C-E). No significant changes in body weight were observed in any group of mice (Fig. S4F). To further define the immune dependency of NAT10 inhibition, CD8⁺ T cell was depleted in tumor bearing mice (Fig. S4G). Flow cytometric analysis demonstrated highly efficient CD8⁺ T cell depletion, with clearance exceeding 95% in peripheral blood (Fig. S4H). Following CD8⁺ T cell depletion, tumor growth in the shNAT10 group no longer differed significantly from the control group (Fig. S4I-K). The body weights of mice in all groups remained stable, with no significant changes observed (Fig. S4L). A similar pattern was observed in final tumor weights. These findings demonstrate that the antitumor efficacy of NAT10 inhibition is mediated predominantly through CD8⁺ T cell dependent immune responses.

Based on these in vivo findings, we next sought to validate whether similar associations exist in clinical specimens. IHC analysis of clinical breast cancer tissues revealed an inverse correlation between PD-L1 expression and CD8⁺ T cell infiltration as well as GzmB⁺ cytotoxic activity (Fig. 9I-J). In addition, the TCGA-BRCA dataset analysis showed that patients with higher expression levels of NAT10, HDAC4, or PD-L1 exhibited a tendency toward increased Treg infiltration (Fig. S4M). Collectively, these results indicated that NAT10 and HDAC4 may facilitate immune evasion in breast cancer by upregulating PD-L1, thereby impairing cytotoxic T cell mediated antitumor responses.

## Discussion

**Fig. 10:**
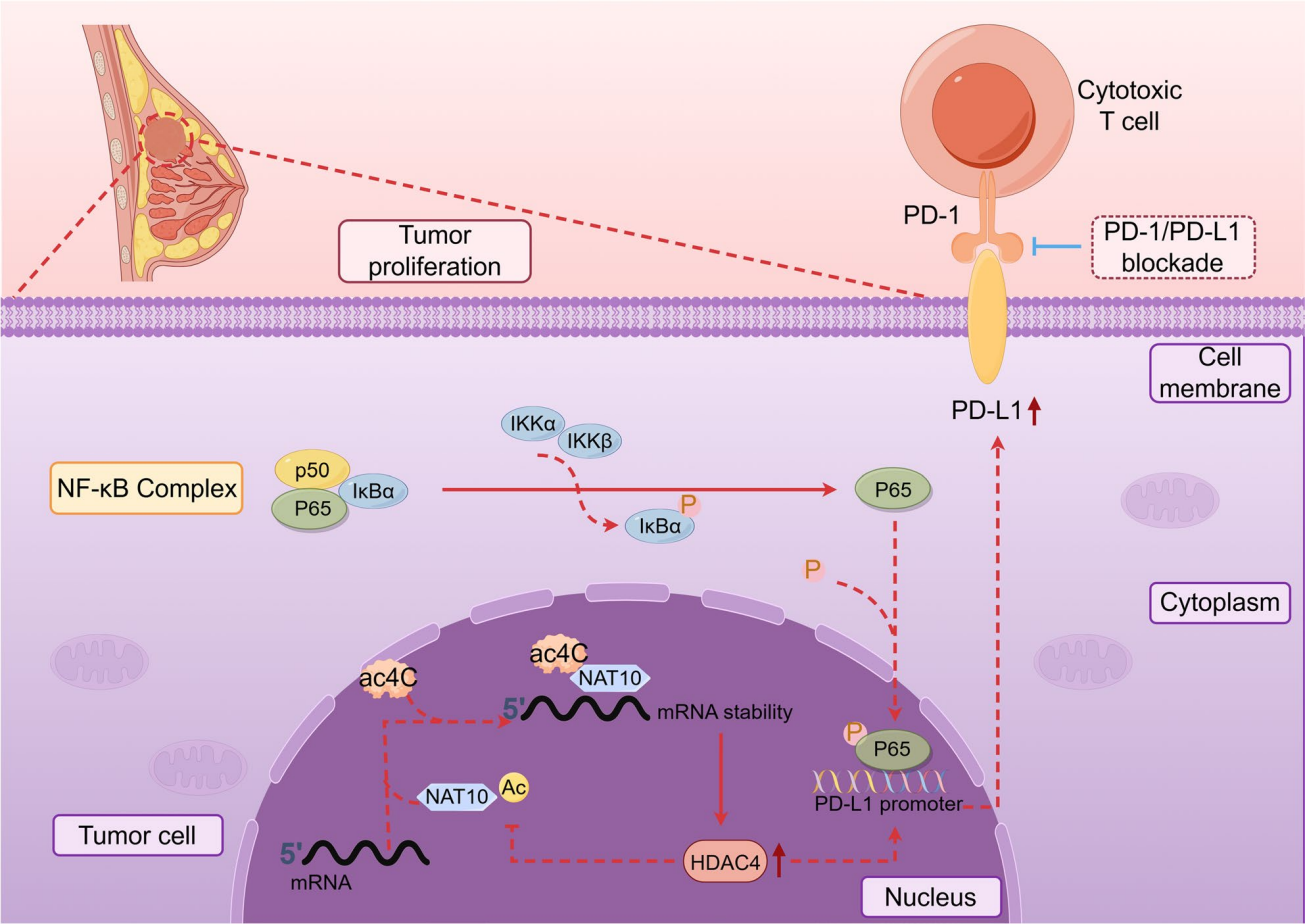
Mode pattern of the NAT10/HDAC4/NF-κB regulatory network in breast cancer. NAT10 mediated ac4C modification stabilizes HDAC4 mRNA, while HDAC4 stabilizes NAT10 protein, forming a reciprocal regulatory loop. HDAC4 activates NF-κB signaling, leading to PD-L1 upregulation and immune evasion. Inhibition of the NAT10/HDAC4/NF-κB axis reduces PD-L1 expression and restores antitumor immunity in breast cancer

RNA modifications are crucial for regulating molecular functions, and pathways governed by these modifications can be exploited for cancer therapy [6]. An increasing number of studies have demonstrated that ac4C modification, as a novel RNA modification, holds great therapeutic potential, making NAT10 a promising target for the treatment of various tumors [13, 31]. Our study revealed that NAT10 mediated ac4C modification is a key driver of breast cancer progression by enhancing the stability of HDAC4 mRNA, which in turn activates the NF-κB pathway and elevates PD-L1 expression, thereby enabling tumor immune escape. Moreover, we identified that HDAC4 post-translationally stabilized NAT10 through deacetylation, forming a self-reinforcing axis that amplifies oncogenic signaling. Together, these findings uncovered a novel link between RNA acetylation and epigenetic-immunological regulation, underscoring the therapeutic potential of targeting the NAT10/HDAC4/NF-κB axis to alleviate immunosuppression in breast cancer (Fig. 10).

As the only known ac4C “writer,” NAT10 mediated ac4C modification plays an essential role in tumor progression by enhancing mRNA stability and translational efficiency. Previous studies have shown that NAT10 mediated ac4C modification of ferroptosis suppressor protein 1 (FSP1) mRNA suppresses ferroptosis, thereby promoting colorectal cancer progression [32]. In prostate cancer, NAT10 stabilizes keratin 8 (KRT8) through ac4C modification, facilitating epithelial mesenchymal transition (EMT) and cell cycle progression, ultimately driving malignancy [33]. Similarly, Helicobacter pylori–induced NAT10 stabilizes mouse double minute 2 (MDM2) mRNA through ac4C modification, promoting gastric cancer progression [34]. In our prior work, we demonstrated that NAT10 stabilizes RAD51 mRNA through ac4C modification, enhancing homologous recombination repair and conferring resistance to poly(ADP-ribose) Polymerase (PARP) inhibitors [35]. These findings establish NAT10 mediated ac4C modification as a broadly oncogenic mechanism across diverse cancer types. Consistent with these observations, we first demonstrated that NAT10 is highly expressed in breast cancer and correlates with poor prognosis. Notably, our study identified HDAC4 as a critical downstream target, suggesting that NAT10 may regulate distinct downstream pathways in a tissue specific manner.

Histone deacetylases (HDACs) were initially regarded as transcriptional repressors due to their ability to deacetylate lysine residues in the N-terminal regions of core histones, thereby altering chromatin accessibility and inhibiting the transcription of specific genes [36–39]. In addition to their effects on histones, HDACs also regulate the acetylation status of non-histone substrates, influencing biological processes associated with tumor progression [40–42]. For example, in breast cancer, the loss of HDAC5 induces the expression of cell cycle genes, leading to resistance to cyclin dependent kinase 4/6 (CDK4/6) inhibitors [43]. Furthermore, HDAC5 has been shown to deacetylate SOX9, promoting c-Myc expression and contributing to resistance to endocrine therapy in ER-positive breast cancer [44]. HDAC1, HDAC2, and HDAC8 induce EMT in breast cancer cells through distinct pathways to facilitate cell migration [45, 46]. HDAC2 and HDAC3 have been shown to suppress vascular endothelial growth factor (VEGF) signaling, which drives angiogenesis to support tumor progression [47]. Inhibition of HDAC2 prevents the nuclear translocation of PD-L1 by maintaining acetylation at lysine 263, thus disrupting its function as a transcription factor that regulates other immune checkpoints [48]. Our study uncovered a reciprocal feedback circuit in which HDAC4, through its deacetylase activity, stabilizes NAT10 protein, thereby reinforcing NAT10 driven oncogenic signaling. This reciprocal regulation represents a unique molecular circuit linking the epitranscriptome and epigenetic regulation.

T cell exhaustion is a well-recognized phenomenon in tumors, in which dysregulation of immune checkpoint proteins leads to T cell dysfunction, a process closely associated with tumor recurrence and progression. This phenomenon has also been proposed as a potential predictor of clinical outcomes [49–51]. However, the mechanisms underlying T cell exhaustion remain incompletely understood. The interaction between PD-1 on T cells and its ligand PD-L1 on tumor cells is particularly critical for inducing T cell dysfunction and exhaustion, representing a major mechanism of tumor immune evasion [52–55]. In this study, we provided novel evidence that NAT10 regulates PD-L1 expression in breast cancer cells through the ac4C/HDAC4/NF-κB signaling pathway. Inhibition of NAT10 enhanced CD8⁺ T cell mediated cytotoxicity against tumor cells, further supporting the role of NAT10 as a critical modulator of CD8⁺ T cell function via the PD-1/PD-L1 axis. These findings are consistent with our previous work, which demonstrated that NAT10 influences cytotoxic T lymphocyte-associated protein 4 (CTLA-4) surface expression on T cells through the ac4C/JunB/LDHA pathway, ultimately playing a role in the establishment of an immunosuppressive microenvironment [27]. Notably, our results are also in agreement with other studies implicating NAT10 in immune regulation across various tumor types. For example, in cervical cancer, the NAT10/ac4C/FOXP1 axis modulates regulatory T cell infiltration by reprogramming glycolytic metabolism, leading to foster an immunosuppressive environment [18]. In pancreatic cancer, NAT10 regulates PD-L1 expression through the LAMB3/FAK/ERK pathway, further highlighting its pleiotropic role in shaping the tumor immune landscape [56]. Moreover, inhibition of tumor intrinsic NAT10 has been shown to activate a type I interferon response via the MYC/CDK2/DNMT1 pathway, which is associated with enhanced antitumor immunity [57]. Although the molecular mechanisms described in these studies differ, they collectively underscore the pivotal role of NAT10 in promoting immune evasion by modulating the tumor immune microenvironment.

The NF-κB pathway is a central regulator of inflammation and immune responses, and its activation is well known to drive PD-L1 transcription, which facilitates tumor immune evasion [58, 59]. In hepatocellular carcinoma, ependymin related 1 (EPDR1) stabilizes IKKβ, a key kinase in the NF-κB pathway, leading to sustained NF-κB activation and subsequent PD-L1 transcription [60]. Our findings complement these reports by demonstrating that HDAC4 promotes PD-L1 expression via the NF-κB pathway, with NAT10 further amplifying this effect. This observation is consistent with studies suggesting that Class I HDAC inhibitors, such as Entinostat, can enhance cytotoxic T lymphocyte activation, inhibit the immunosuppressive activities of Tregs and MDSCs, and reverse the immunosuppressive tumor microenvironment [61]. Taken together, our study underscores the therapeutic potential of targeting the NAT10/ac4C/HDAC4/NF-κB signaling axis in breast cancer. Such an approach may represent a novel strategy to enhance the efficacy of immune checkpoint inhibitors and provide insights for developing combination immunotherapies aimed at overcoming immune escape in cancer.

Several limitations should be acknowledged in this study. Our analyses predominantly relied on cell lines and mouse subcutaneous tumor models, which may not fully replicate the complexity of the human tumor microenvironment. The potential of NAT10 inhibition to influence tumorigenesis requires further validation in immunocompetent models and clinical settings. Moreover, although the tested regimens were well tolerated in our in vivo studies, further long-term investigations with higher dose are needed to fully evaluate their toxicological profile and potential for preclinical clinical application. The tumor immune microenvironment comprises diverse cell types, and although this study primarily focused on tumor cell–intrinsic mechanisms, the effects of NAT10 inhibition on other immune cell subsets and their interactions remain to be elucidated. Furthermore, it is important to recognize that multiple HDAC family members can act on common substrates, including transcription factors and cyclins. Thus, inhibition of HDAC4 may not completely abolish substrate deacetylation, as other HDACs could compensate and maintain their functions. This highlights the necessity of excluding potential confounding effects of other HDACs in future investigations.

## Conclusions

In conclusion, our study identified the NAT10/ac4C/HDAC4 regulatory axis as a fundamental driver of breast cancer progression and immune evasion. The reciprocal stabilization loop between NAT10 and HDAC4 established a persistent oncogenic signal that overactivated NF-κB and induced PD-L1 expression. Inhibition of this axis offers a promising therapeutic strategy with dual effects: direct antitumor activity and restoration of antitumor immunity. Future studies should focus on elucidating additional molecular mechanisms, exploring broader immunological effects, validating the efficacy and safety of combination therapies in immunocompetent models, and evaluating the biomarker potential of this axis to guide patient selection for targeted immunotherapies. Assessing NAT10 expression levels may provide valuable insights for immunotherapy application, and optimizing treatment strategies could ultimately improve patient prognosis.

## Supplementary Information

Supplementary Material 1.

Supplementary Material 2.

Supplementary Material 3.

Supplementary Material 4.

Supplementary Material 5.

## Data Availability

The datasets used and analyzed during the current study are available from the corresponding author on reasonable request. The sequencing data have been deposited in the NCBI BioProject database under accession numbers PRJNA1381095, PRJNA1381081, and PRJNA1381076.

## Abbreviations

NAT10: N-acetyltransferase 10
ac4C: N4-acetylcytidine
RIP: RNA immunoprecipitation
acRIP: ac4C RNA immunoprecipitation
Co-IP: Co-Immunoprecipitation
HDAC4: Histone deacetylase 4
PD-L1: Programmed death-ligand 1
GNAT: GCN5-related N-acetyltransferase
KIF23: Kinesin family member 23
HIF-1: hypoxia inducible factor 1
SEPT9: Septin 9
ITGB5: Integrin beta 5
IHC: Immunohistochemistry
OS: Overall survival
DMEM: Dulbecco’s Modified Eagle Medium
RPMI 1640: Roswell Park Memorial Institute 1640 medium
L-15: Leibovitz’s L-15
siRNA: small interfering RNA
shRNA: short hairpin RNA
qRT-PCR: quantitative reverse transcription PCR
PI: propidium iodide
cDNA: complementary DNA
BCA: Bicinchoninic acid
PVDF: Polyvinylidene fluoride
HE: Hematoxylin & Eosin
BSA: Bovine serum albumin
IF: Immunofluorescence
ChIP: Chromatin immunoprecipitation
SPF: Specific pathogen free
GzmB: Granzyme B
SD: Standard deviation
TCGA: The Cancer Genome Atlas
CDS: Coding sequence
3′ UTR: 3′ untranslated region
GO-BP: Gene Ontology-biological process
KEGG: Kyoto Encyclopedia of Genes and Genomes
DEGs: differentially expressed genes
WT: Wild-type
MUT: ac4C site mutant
PD-1: Programmed death-1
GSEA: Gene set enrichment analysis
NES: Normalized enrichment score
FSP1: Ferroptosis suppressor protein 1
KRT8: Keratin 8
EMT: Epithelial mesenchymal transition
MDM2: Mouse double minute 2
PARP: Poly(ADP-ribose) Polymerase
HDACs: Histone deacetylases
CDK4/6: Cyclin dependent kinase 4/6
VEGF: Vascular endothelial growth factor
CTLA-4: Cytotoxic T lymphocyte-associated protein 4
EPDR1: Ependymin related 1
