# Supplementary material for: Targeting the NAT10-HDAC4 positive feedback loop counteracts immunosuppression in breast cancer
Source: J Exp Clin Cancer Res. 2026 Jan 7;45:35. doi: 10.1186/s13046-025-03638-7 (PMC12870259; doi:10.1186/s13046-025-03638-7)
Supplement: Supplementary file 1 — Supplementary Material 1. [file 13046_2025_3638_MOESM1_ESM.docx]

**Supplementary Figures**


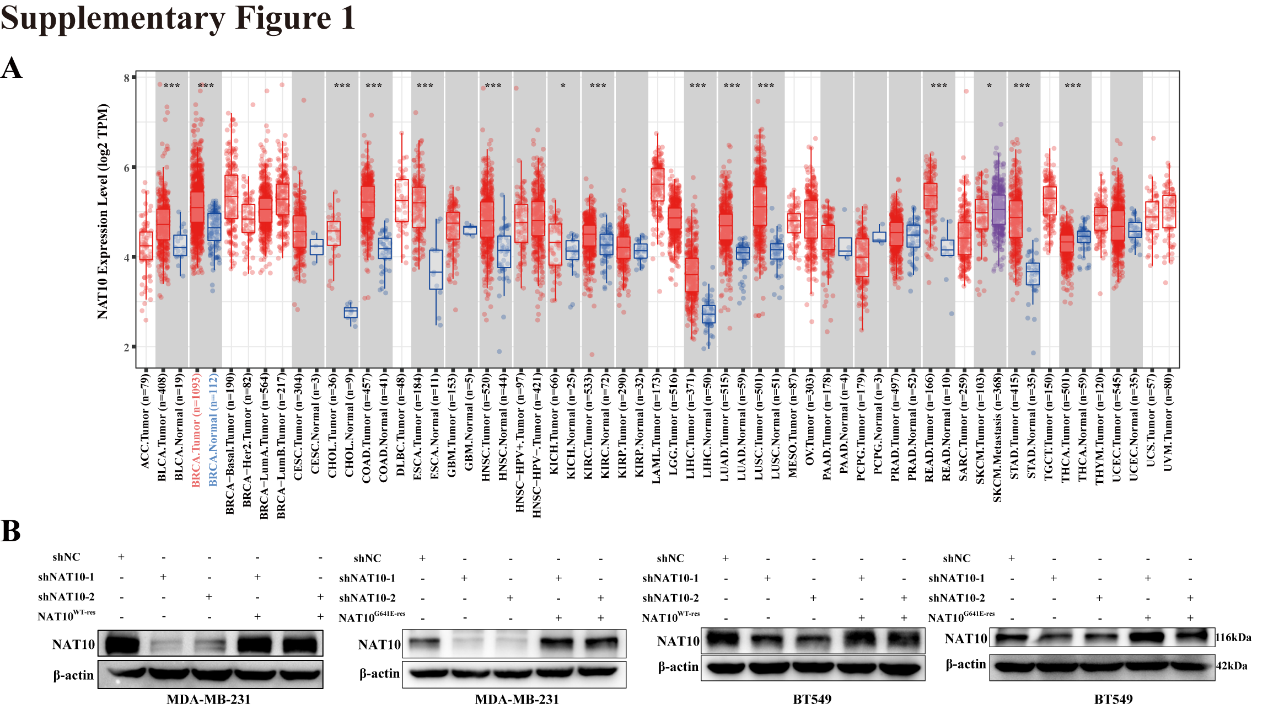


**Figure S1. NAT10 mediated ac4C modification promotes breast cancer progression.**

1. Pan-cancer analysis of NAT10 expression using the TIMER 2.0 database.
2. Western blot analysis of NAT10 protein levels in MDA-MB-231 and BT549 cells transfected with shNC, shNAT10-1, or shNAT10-2, with or without rescue by NAT10^WT-res^ or NAT10^G614E-res^. All data are presented as mean ± SD. * *P* < 0.05, ** *P* < 0.01, *** *P* < 0.001, **** *P* < 0.0001.

**
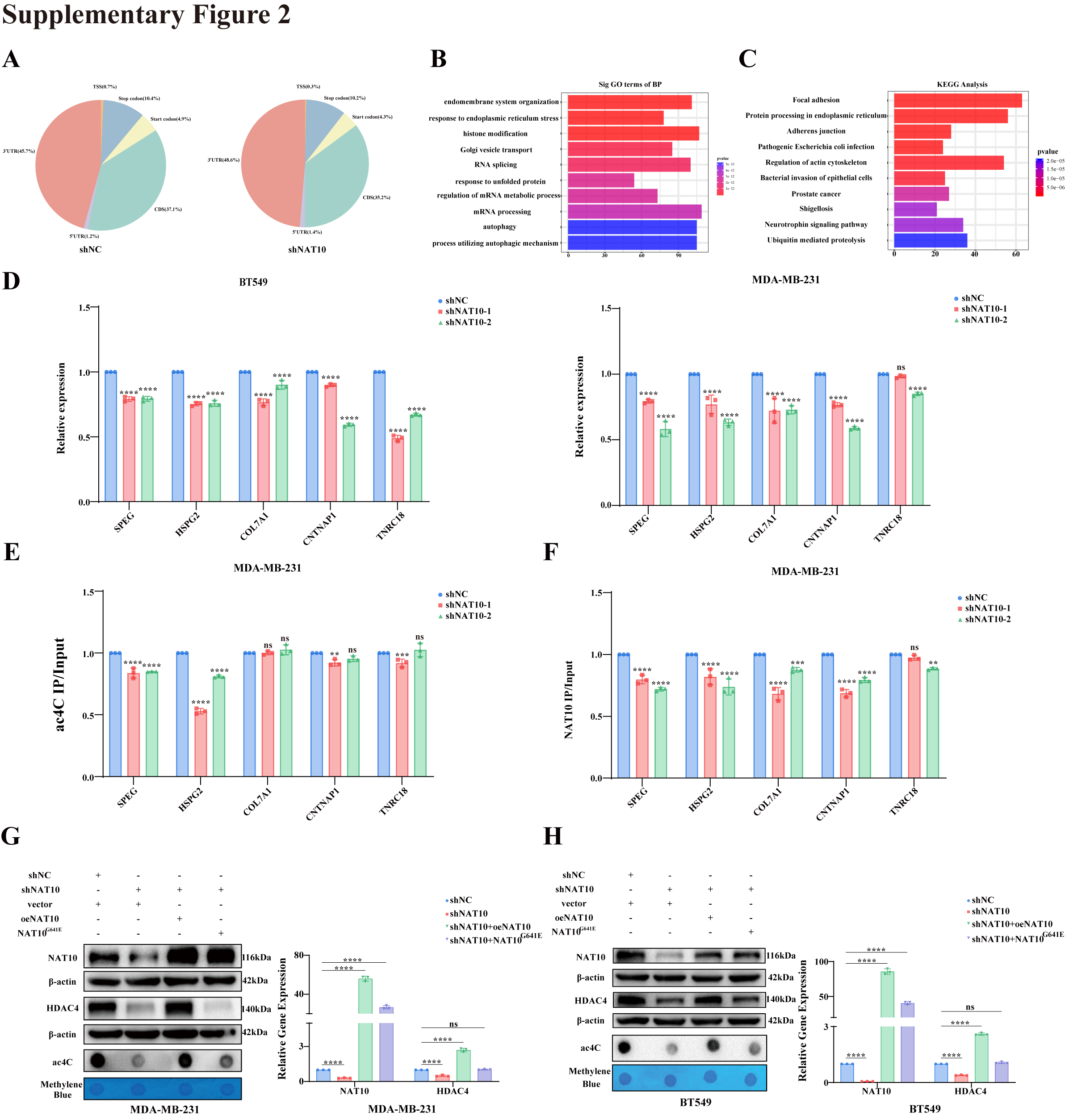
**

**Figure S2. NAT10 stimulates HDAC4 expression via ac4C modification. (A)** Distribution of ac4C peaks predominantly within CDS and 3′UTR regions. **(B-C)** GO (**B**) and KEGG pathway (**C**) analyses of genes with differential ac4C modification identified by acRIP-seq. **(D)** Relative gene expression measured by qRT-PCR upon NAT10 knockdown. **(E-F)** acRIP-qPCR analysis of ac4C modification **(E)** and RIP-qPCR analysis of NAT10 binding to target mRNAs **(F)** following NAT10 knockdown. **(G-H)** Relative HDAC4 expression assessed by qRT-PCR, Western blot, and dot blot after transfection with shNC, shNAT10, shNAT10 plus oeNAT10, or shNAT10 plus NAT10^G641E^. All data are presented as mean ± SD. * *P* < 0.05, ** *P* < 0.01, *** *P* < 0.001, **** *P* < 0.0001.


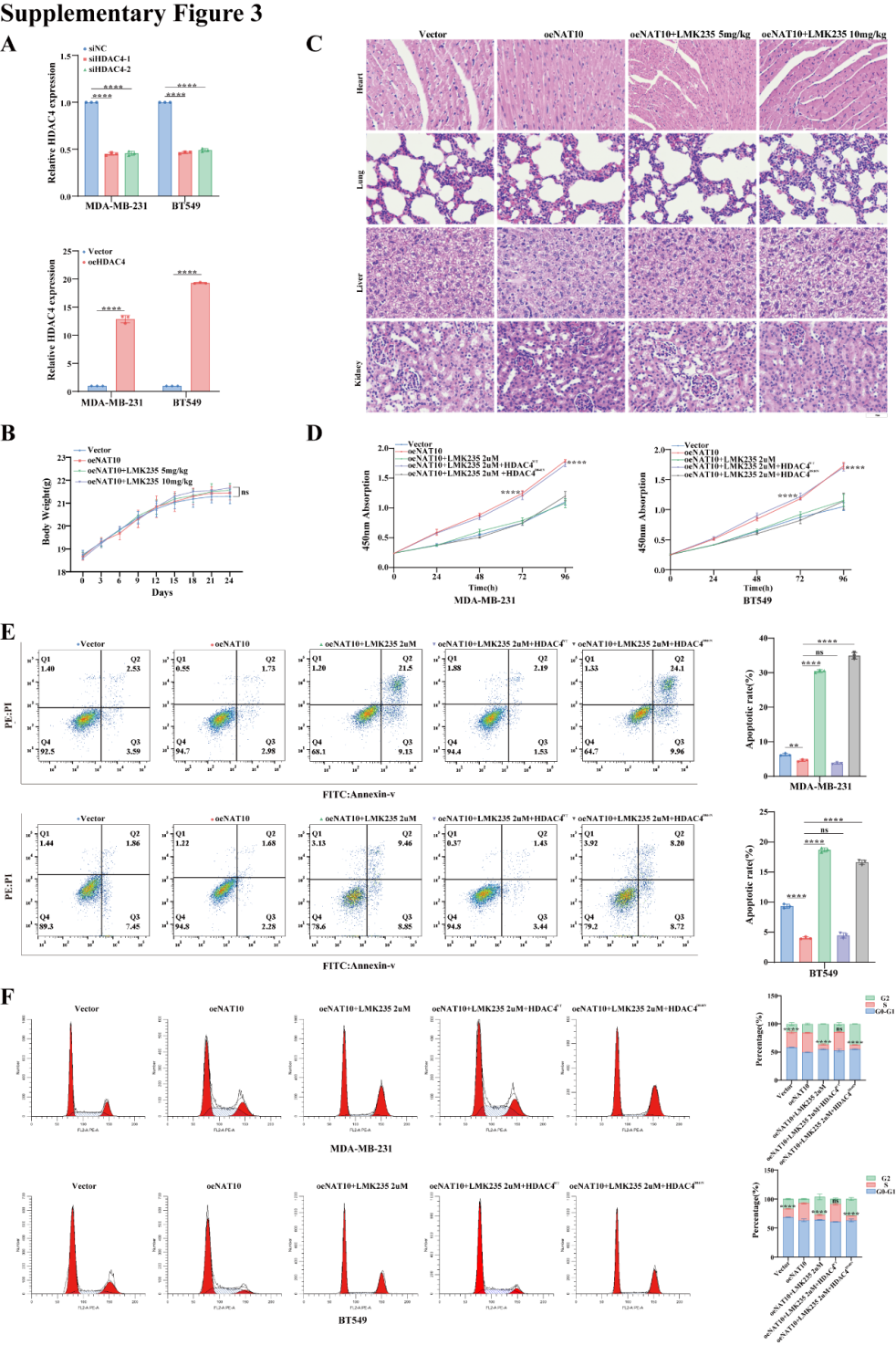


**Figure S3. The proliferative function of NAT10 in breast cancer depends on HDAC4. (A)** Relative HDAC4 expression assessed by qRT-PCR after HDAC4 knockdown or overexpression. **(B)** Body weight curves of mice treated with vector, oeNAT10, oeNAT10 plus LMK235 (5 mg/kg), or oeNAT10 plus LMK235 (10 mg/kg) (*n* = 6). **(C)** Representative H&E images of the heart, lung, liver, and kidney from mice treated with vector, oeNAT10, oeNAT10 plus LMK235 (5 mg/kg), or oeNAT10 plus LMK235 (10 mg/kg). (*n* = 6; scale bars, 50 μm). **(D-F)** CCK-8 assays **(D)** and flow cytometry analyses of apoptotic rates **(E)** and cell cycle distribution **(F)** in cells transfected with vector, oeNAT10, oeNAT10 plus LMK235 (2 µM), oeNAT10 plus LMK235 (2 µM) plus HDAC4^WT^, or oeNAT10 plus LMK235 (2 µM) plus HDAC4^D840N^. All data are presented as mean ± SD. * *P* < 0.05, ** *P* < 0.01, *** *P* < 0.001, **** *P* < 0.0001.

**
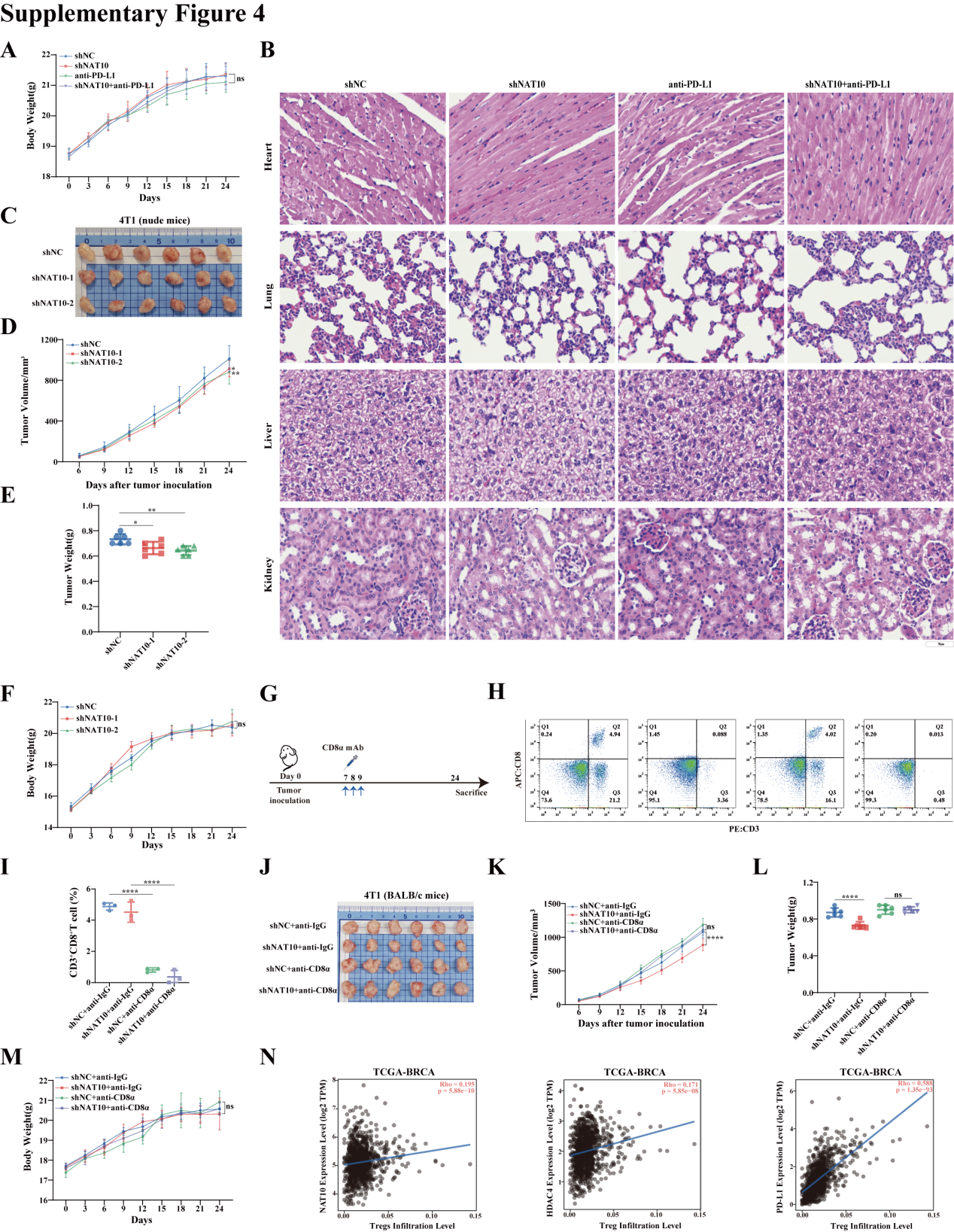
**

**Figure S4. Inhibition of NAT10 enhances the efficacy of anti-PD-L1 therapy *in vivo*. (A)** Body weight curves of mice treated with shNC, shNAT10, anti-PD-L1 antibody, or the combination of shNAT10 and anti-PD-L1 (*n* = 6). **(B)** Representative H&E staining of the heart, lung, liver, and kidney from mice bearing 4T1 tumors across different treatment groups (*n* = 6; scale bars, 50 μm). **(C-F)** Tumor growth in nude mice bearing shNC or shNAT10 4T1 tumors (*n* = 6), including representative tumor images **(C)**, tumor growth curves **(D)**, tumor weights **(E)**, and body weight curves **(F)**. **(G)** Schematic illustration of the tumor model and treatment schedule with anti-CD8α antibody administration. **(H-I)** Representative flow cytometry plots showing CD3⁺CD8⁺ T cells (PE-CD3 vs. APC-CD8) in the peripheral blood of mice bearing shNC or shNAT10 tumors treated with IgG or anti-CD8α antibody, together with quantification of CD3⁺CD8⁺ T cells frequencies. **(J-M)** Representative tumor images **(J)**, tumor growth curves **(K)**, tumor weights **(L)**, and body weight curves **(M)** of 4T1 tumor bearing mice treated with shNC plus anti-IgG, shNAT10 plus anti-IgG, shNC plus anti-CD8α, or shNAT10 plus anti-CD8α (*n* = 6). **(N)** Correlation analysis of NAT10, HDAC4, and PD-L1 mRNA expression with Treg infiltration levels in the TCGA RNA-seq dataset.

All data are presented as mean ± SD. * *P* < 0.05, ** *P* < 0.01, *** *P* < 0.001, **** *P* < 0.0001.
